# Investigations into Hypoxia and Oxidative Stress at the Optic Nerve Head in a Rat Model of Glaucoma

**DOI:** 10.3389/fnins.2017.00478

**Published:** 2017-08-24

**Authors:** Glyn Chidlow, John P. M. Wood, Robert J. Casson

**Affiliations:** Ophthalmic Research Laboratories, Discipline of Ophthalmology and Visual Sciences, University of Adelaide Adelaide, SA, Australia

**Keywords:** glaucoma, optic nerve head, axonal transport, hypoxia, oxidative stress, astrocyte, retinal ganglion cell

## Abstract

The vascular hypothesis of glaucoma proposes that retinal ganglion cell axons traversing the optic nerve head (ONH) undergo oxygen and nutrient insufficiency as a result of compromised local blood flow, ultimately leading to their degeneration. To date, evidence for the hypothesis is largely circumstantial. Herein, we made use of an induced rat model of glaucoma that features reproducible and widespread axonal transport disruption at the ONH following chronic elevation of intraocular pressure. If vascular insufficiency plays a role in the observed axonal transport failure, there should exist a physical signature at this time point. Using a range of immunohistochemical and molecular tools, we looked for cellular events indicative of vascular insufficiency, including the presence of hypoxia, upregulation of hypoxia-inducible, or antioxidant-response genes, alterations to antioxidant enzymes, increased formation of superoxide, and the presence of oxidative stress. Our data show that ocular hypertension caused selective hypoxia within the laminar ONH in 11/13 eyes graded as either medium or high for axonal transport disruption. Hypoxia was always present in areas featuring injured axons, and, the greater the abundance of axonal transport disruption, the greater the likelihood of a larger hypoxic region. Nevertheless, hypoxic regions were typically focal and were not necessarily evident in sections taken deeper within the same ONH, while disrupted axonal transport was frequently encountered without any discernible hypoxia. Ocular hypertension caused upregulation of heme oxygenase-1—an hypoxia-inducible and redox-sensitive enzyme—in ONH astrocytes. The distribution and abundance of heme oxygenase-1 closely matched that of axonal transport disruption, and encompassed hypoxic regions and their immediate penumbra. Ocular hypertension also caused upregulations in the iron-regulating protein ceruloplasmin, the anaerobic glycolytic enzyme lactate dehydrogenase, and the transcription factors cFos and p-cJun. Moreover, ocular hypertension increased the generation of superoxide radicals in the retina and ONH, as well as upregulating the active subunit of the superoxide-generating enzyme NADPH oxidase, and invoking modest alterations to antioxidant-response enzymes. The results of this study provide further indirect support for the hypothesis that reduced blood flow to the ONH contributes to axonal injury in glaucoma.

## Introduction

Glaucoma, the leading cause of irreversible blindness worldwide (Quigley and Broman, 2006), encompasses a family of neurodegenerative diseases, all of which feature a clinically characteristic optic neuropathy (Casson et al., 2012). Despite significant progress in recent decades, the pathogenesis of glaucoma remains poorly understood, while therapeutic options are restricted to reducing intraocular pressure (IOP), the foremost treatable risk factor for the disease. In rodent (Howell et al., 2007; Crish et al., 2010; Salinas-Navarro et al., 2010; Chidlow et al., 2011b; Dengler-Crish et al., 2014), as well as primate (Anderson and Hendrickson, 1974; Quigley and Anderson, 1976; Minckler et al., 1977), models of glaucoma, failure of orthograde axonal transport has emerged as the earliest detectable pathological event. While the primary site of axonal injury in glaucoma has not been unequivocally identified, data from clinical, as well as animal, studies have highlighted the crucial role played by the optic nerve head (ONH)—the location where retinal ganglion cell (RGC) axons converge to form the optic nerve and traverse the lamina—in this process. In simple terms, two theories have been proposed to account for axonal injury at the ONH in glaucoma: “the mechanical” and “the vascular” theories (Fechtner and Weinreb, 1994).

The vascular hypothesis proposes that RGC axons passing through the ONH undergo chronic or intermittent hypoxia, ischemia, and/or hypoglycemia as a result of compromised local blood flow (Flammer et al., 2002). The unmyelinated axons of the ONH are thought to be highly vulnerable to a decreased oxygen/nutrient supply owing to their prodigious energy requirements, which are served by a high local density of mitochondria (Barron et al., 2004). Deficits in nutrients or oxygen will not only result in RGC axons becoming bioenergetically compromised (Inman and Harun-Or-Rashid, 2017), but, importantly, will also dramatically increase production of reactive oxygen species (ROS), leading to oxidative stress (Chrysostomou et al., 2013). The relationship between ischemia-reperfusion and oxidative stress is well-known (Chen et al., 2011). It seems paradoxical that low oxygen availability *per se* would also result in an increase in ROS; however, numerous studies have reported that hypoxia increases generation of ROS within complex III of mitochondria (see Guzy and Schumacker, 2006). The rationale for this phenomenon is that during hypoxia, mitochondrial electron transport slows, augmenting the reduction state of electron carriers. This accumulation of reducing equivalents favors superoxide production at low oxygen concentrations. Thus, reperfusion is not essential for oxidative stress. Oxidative stress itself represents the failure of endogenous antioxidant defenses, which comprise both enzymatic and non-enzymatic components, to efficiently detoxify oxidative free radical species. Cumulative oxidative stress causes damage to DNA, proteins and lipids.

There is a wealth of physiological data supporting blood flow incompetence at the ONH in glaucoma individuals (Satilmis et al., 2003; Schmidl et al., 2011; Yanagi et al., 2011); however, direct evidence for the presence of ischemia/hypoxia within the ONH is predictably sparse. Nevertheless, there is an increasing body of indirect evidence for the vascular hypothesis, which includes reports of increases in oxidative stress markers in the serum, aqueous humor, and retina of glaucoma patients (Farkas et al., 2004; Aslan et al., 2013; Benoist d'Azy et al., 2016). These studies suggest a general compromise of antioxidant defenses in glaucoma. Interestingly, oxidative stress-related events have also been documented in ONH astrocyte and lamina cribrosa cell cultures from glaucoma patients (Malone and Hernandez, 2007; McElnea et al., 2011). In rodent models of glaucoma, convincing evidence exists for enhanced ROS levels and increased oxidative stress within the retina (see for example Moreno et al., 2004; Ko et al., 2005; Inman et al., 2013); somewhat surprisingly, however, the ONH itself has received scant attention.

In the current study, we made use of an induced rat model of glaucoma that features reproducible and widespread axonal transport disruption at the ONH by 24 h after chronic elevation of IOP (Salinas-Navarro et al., 2010; Chidlow et al., 2011b). It has been postulated that the model precipitates a crush-like injury to ON axons (Vidal-Sanz et al., 2011); however, if vascular insufficiency at the ONH—caused by ocular hypertension (OHT)—plays a role in the observed axonal transport failure, there should be a physical signature at this time point. Using a range of immunohistochemical and molecular tools, we looked for cellular events indicative of vascular insufficiency, including the presence of hypoxia, upregulation of hypoxia-inducible or antioxidant-response genes, alterations to antioxidant enzymes, increased formation of superoxide, upregulation in ROS-generating NADPH oxidase, and the presence of oxidative stress.

## Materials and methods

### Animals and procedures

This study was approved by the South Australia Pathology/Central Health Network Animal Ethics committee and conforms with the Australian Code of Practice for the Care and Use of Animals for Scientific Purposes, 2013. All procedures were performed under anesthesia (100 mg/kg ketamine and 10 mg/kg xylazine), and all efforts were made to minimize suffering. All experiments also conformed to the ARVO Statement for the Use of Animals in Ophthalmic and Vision Research. Adult Sprague-Dawley rats (220–300 g) were housed in a temperature- and humidity-controlled environment with a 12-h light, 12-h dark cycle, and were provided with food and water *ad libitum*.

Ocular hypertension was induced in the right eye of each animal by laser photocoagulation of the trabecular meshwork, as previously described (Ebneter et al., 2010). IOPs were measured in both eyes using a rebound tonometer, factory calibrated for use in rats. All animals demonstrated an adequate IOP elevation (minimum increase in IOP of 10 mmHg). One animal was excluded as a result of death under anesthesia and 1 due to hyphema. The number of rats analyzed for immunohistochemistry was as follows: 1 d time point, *n* = 28; 3 d time point, *n* = 16. Of the total number of rats analyzed at 1 d, *n* = 16 received an injection of pimonidazole for localization of regions of hypoxia (see below), while *n* = 4 received an injection of dihydroethidium for localization of superoxide radicals (see below). Of the total number of rats analyzed at 3 d, *n* = 4 received an injection of dihydroethidium for localization of superoxide. In addition to animals used for immunohistochemistry, a further 14 (1 d time point) and 12 (3 d time point) rats were analyzed by qPCR/Western blotting. In all cases throughout the manuscript, “*n*” number refers to the number of animals analyzed.

### Tissue processing and immunohistochemistry

For tissue harvesting of ONH for protein and RNA extraction, all rats were humanely killed by transcardial perfusion with physiological saline under terminal anesthesia. Eyes were immediately enucleated and ONH samples were prepared using the following method: the anterior portion and vitreous body from each eye were removed. The remaining eye-cup was subsequently dissected into a flattened whole-mount in the shape of a “maltese-cross.” A biopsy punch of 2 mm in diameter (Stiefel Laboratories, Brentford, United Kingdom, cat # BIOPSY-5918) was then utilized to separate the ONH area from the remainder of the ocular tissue. The initial 1 mm length of optic nerve was also included within each sample, as was the very central central portion of the retina. ONH samples were placed in 400 μl of TRI-reagent and then sonicated. Subsequently, both total protein and total RNA were extracted.

For tissue harvesting for paraffin embedding, all rats were killed by transcardial perfusion with physiological saline under terminal anesthesia. Eyes were immediately enucleated and immersion fixed in 10% (w/v) neutral buffered formalin or, in some cases, in Davidson's fixative [22% formalin (37–40%) solution, 33% ethanol (95%), 11.5% glacial acetic acid] for 24 h, followed by routine processing for paraffin embedding. Eyes were marked in a specific and recorded location to ensure correct orientation during embedding and 4 μm serial sections were cut using a rotary microtome.

Colorimetric immunohistochemistry was performed as previously described (Chidlow et al., 2011a). Briefly, tissue sections were deparaffinized and endogenous peroxidase activity was blocked with H_2_O_2_. Antigen retrieval was performed by microwaving sections in 10 mM citrate buffer (pH 6.0) and non-specific labeling blocked with PBS containing 3% normal horse serum. Sections were incubated overnight at room temperature in primary antibody (see Table 1), followed by consecutive incubations with biotinylated secondary antibody (Vector, Burlingame, CA) and streptavidin-peroxidase conjugate (Pierce, Rockford, IL). Color development was achieved using NovaRed substrate kit (Vector, Burlingame, CA) for 3 min. Sections were counterstained with hematoxylin, dehydrated, cleared in histolene, and mounted. Confirmation of the specificity of antibody labeling was judged by the morphology and distribution of the labeled cells, by the absence of signal when the primary antibody was replaced by isotype/serum controls, by comparison with the expected staining pattern based on our own, and other, previously published results, and, in some instances, by the detection within retinal samples of a protein at the expected molecular weight by Western blotting.

**Table 1:** Primary Antibodies used in the study.

**Protein**	**Source**	**Clone/Cat. No**.	**Species**	**Immunogen**	**Dilution**
8-Hydroxy-2′-deoxyguanosine	Abcam	Cat# ab48508	Mouse	8-Hydroxy-2′-deoxyguanosine conjugated Keyhole Limpet Hemocyanin	1:20,000
Actin	Sigma	Clone AC-15	Mouse	Slightly modified β-cytoplasmic actin N-terminal peptide	1:20,000^W^
APP	^*^C Masters	Clone 22C11	Mouse	Purified recombinant Alzheimer precursor A4 (pre A4695) fusion protein.	1:1,500
Ceruloplasmin	Dako	Cat# Q0121	Rabbit	*Ceruloplasmin* isolated from human plasma	1:10,000^W^
cFOS	Santa-Cruz	Cat# sc-253	Rabbit	Epitope mapping within an internal region of c-Fos of human origin	1:5,000
CRALBP	Abcam	Cat# ab15051	Mouse	Recombinant full length human CRALBP	1:2,000
gp91^phox^	BD Biosciences	Clone 53/gp91[phox]	Mouse	Mouse gp91[phox] aa. 450–556	1:500
HO-1	Enzo Life Sciences	Cat# SPA-895	Rabbit	Recombinant rat HO-1 (Hsp32) lacking the membrane spanning region	1:2,500, 1:2,000^W^
Interleukin-6	R&D Systems	Cat# AF506	Goat	*E. coli*-derived recombinant rat IL-6 Phe25-Thr211	1:500
Iba1	Wako	Cat# 019-19741	Rabbit	Synthetic peptide corresponding to the Iba1carboxy-terminal sequence	1:4,000^f^
LDH-A	Santa-Cruz	Cat# sc-27230	Goat	Epitope mapping at the N-terminus of LDH-A of human origin	1:2,000
Myelin basic protein	Dako	Cat# A0623	Rabbit	*Myelin basic, protein* isolated from human brain	1:3,000
p67^phox^	BD Biosciences	Clone 29/p67phox	Mouse	Human p67 [phox] aa. 317-469	1:1,000^W^
p-cJUN	CST	Cat# 3270	Rabbit	Synthetic phosphopeptide corresponding to residues around Ser73 of human c-Jun	1:5,000
Pimonidazole	Hypoxyprobe Inc	Clone 4.3.11.3	Mouse	Pimonidazole adducts	1:500
SOD-1	Calbiochem	Clone 6F5	Mouse	Purified recombinant fragment of human SOD1 expressed in *E. Coli*	1:2,500
SOD-2	Antibody Technology Australia Pty Ltd	Cat# SOD2R	Rabbit	Human/rat/mouse SOD2 aa. 25–43	1:2,500
Transferrin receptor	ThermoFisher	Clone H68.4	Mouse	Recombinant human transferrin receptor	1:500
Vimentin	Dako	Clone V9	Mouse	Purified vimentin from porcine eye lens	1:200^f^

f Dilution used for 2-step fluorescent immunostaining procedure;

W Dilution used for Western blotting;

* *Gifted by C Masters, University of Melbourne*.

For double labeling fluorescent immunohistochemistry, visualization of one antigen was achieved using a 3-step procedure (primary antibody, biotinylated secondary antibody, streptavidin-conjugated AlexaFluor 488 or 594), while the second antigen was labeled by a 2-step procedure (primary antibody, secondary antibody conjugated to AlexaFluor 488 or 594). Sections were prepared as above, then incubated overnight at room temperature in the appropriate combination of primary antibodies. On the following day, sections were incubated with the appropriate biotinylated secondary antibody for the 3-step procedure plus the correct secondary antibody conjugated to AlexaFluor 488 or 594 for the 2-step procedure, followed by streptavidin-conjugated AlexaFluor 488 or 594. Sections were then mounted using anti-fade mounting medium and examined under a confocal fluorescence microscope.

### Evaluation of immunohistochemistry

**Figure 1:**
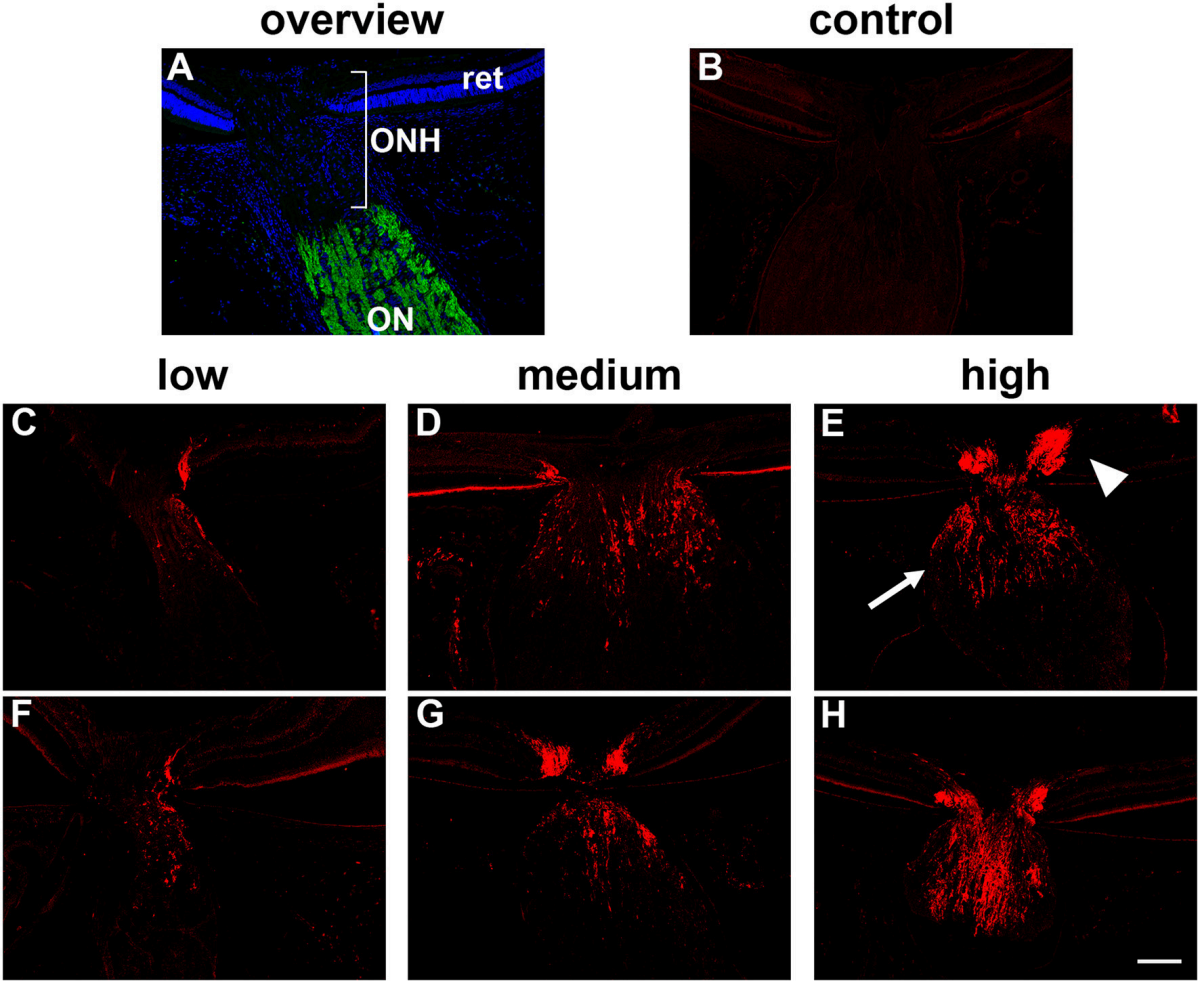
Inter-animal variability in axonal transport disruption at the optic nerve head (ONH) 24 h after induction of ocular hypertension. **(A)** Overview of the anatomy of the rat ONH, as labeled with the nuclear dye DAPI. The location at which RGC axons become myelinated is demarcated by myelin basic protein (green). **(B)** In normal rats, minimal interleukin-6 is associated with RGC axons. **(C–H)** At 24 h after induction of chronic ocular hypertension, accumulation of interleukin-6 is evident within axons in the prelaminar (arrowhead) and laminar (arrow) ONH. The abundance of axonal transport disruption varies markedly between animals, ranging from a few to numerous immunopositive fibers, which can be classified, using a simple scoring system, as low, medium, or high; two examples are shown for each category. ON, optic nerve; ret, retina. Scale bar: 100 μm.

All assessments were performed in a randomized, blinded manner. The presence or absence of a hypoxic region within each pimonidazole-injected rat was compared to the IOP of that rat and also to the magnitude of axonal transport disruption at the ONH. Disrupted axonal transport was assessed semi-quantitatively by immunolabeling either for interleukin-6 (IL-6) or β-Amyloid precursor protein (APP) accumulation throughout the ONH using a 4-point grading system (undetectable, low, medium, high; see Figure 1) as previously described (Chidlow et al., 2011b, 2012). Our previous data have shown that IL-6 and APP can be used interchangeably; they provide identical patterns of immunolabeling within the ONH at 24 h after IOP elevation and identical responses to tissue injury.

For assessment of the amount of heme oxygenase-1 (HO-1), lactate dehydrogenase-A (LDH-A), p-cJun, cFos, transferrin receptor, and NADPH oxidase immunoreactivities in treated and control animals, photomicrographs (350 × 260 μm) of the ONH (centered at ~0.4 mm from the scleral margin), and of the proximal myelinated ON (centered at ~1.4 mm from the scleral margin) were captured from each animal. The area of axonal tissue available for analysis within each ONH typically did not encompass the entire area of the photomicrograph, hence the image was cropped. The corresponding image from the ON of that animal was cropped identically, which permits direct comparison of the % area stained in the ONH vs. the ON. For HO-1, LDH-A, transferrin receptor, and NADPH oxidase, images underwent color deconvolution to eliminate haematoxylin staining. After manual thresholding, the area of positive immunolabeling was measured. Evaluations were performed using the ImageJ 1.42q software package platform (http://rsb.info.nih.gov/ij/) and data are presented as % area of immunolabeling ± SEM. For p-cJun and cFos, the number of cells with unambiguous positive nuclear staining were counted in each photomicrograph. The threshold for identification of positive labeling was performed manually and took into account comparison with template photomicrographs and inter-animal variability in background labeling. Data are presented as number of positive cells ± SEM. In addition, for HO-1, two photomicrographs (350 × 260 μm) of the central retina, taken at ~500 μm from the ONH, were also captured and analyzed for % area of immunolabeling, as outlined above. Statistical analysis (control group vs. OHT group) was carried out by Student's unpaired *t-*test where parametric assumptions were met or Mann–Whitney Rank Sum Test where they were not.

### Localization of hypoxia

To detect cellular hypoxia, 60 mg/kg bodyweight pimonidazole hydrochloride (Hypoxyprobe^*TM*^-1 kit, Hypoxyprobe Inc, Burlington, Massachusetts) diluted in sterile PBS was administered by intraperitoneal injection 3 h prior to killing, as previously described (Gardiner et al., 2005; Mowat et al., 2010). Pimonidazole forms covalent adducts in cells that have an partial pressure of oxygen which is <10 mmHg (Arteel et al., 1995). The subsequent staining of tissue sections with an anti-pimonidazole antibody reveals the presence of hypoxic cells (Holcombe et al., 2008). Rats were killed by transcardial perfusion with physiological saline, following which they were immersion fixed in 10% neutral buffered formalin and processed for paraffin embedding and immunohistochemistry, as described above. In pimonidazole-injected animals, tissue sections from three levels of the ONH were typically evaluated for hypoxia.

### Localization of intracellular superoxide

The spatial production of superoxide was investigated by *in situ* detection of the oxidation product of dihydroethidium (DHE, Molecular Probes, USA). DHE, which is cell permeant, is converted intracellularly to an ethidium derivative, in the presence of superoxide (but not by hydrogen peroxide, hydroxyl radical or peroxynitrite). This ethidium derivative exhibits peak fluorescence in the red spectrum and binds to DNA (Zanetti et al., 2005). Five microliters of DHE solution (stock solution of 5 mM in dimethyl sufoxide, diluted to 200 μM in PBS) was administrated by intravitreal injection to both eyes 3 h prior to humane killing. Assuming a volume of vitreous humor of 100 μl, this equated to a final DHE concentration of ~10 μM. Rats were killed by transcardial perfusion with physiological saline followed by 10% (w/v) neutral buffered formalin. After post-fixation overnight, also in 10% (w/v) neutral buffered formalin, 10 μm sagital sections were prepared on a cryostat, rinsed with PBS and then mounted in fluorescence-preserving mounting medium. Slides were photographed using a confocal fluorescence microscope with an excitation of 510–550 nm and an emission of >580 nm.

### Real-time RT-PCR

Real time PCR (qPCR) studies were carried out essentially as described previously (Chidlow et al., 2008). In brief, tissues were dissected, total RNA was isolated and first strand cDNA was synthesized from DNase-treated RNA samples. Real-time PCR reactions were carried out in 96-well optical reaction plates using the cDNA equivalent of 10 ng total RNA for each sample in a total volume of 20 μl containing 1 × SYBR Green or 1 × SSO Advanced PCR master mix (BioRad, Gladesville, Australia), forward and reverse primers. Thermal cycling conditions were 95°C for 3 min followed by 40 cycles of amplification comprising 95°C for 12 s, appropriate annealing temperature for 30 s and 72°C for 30 s. Primer pairs (Table 2) were designed from sequences contained in the Genbank database using the primer design software Primer 3 (http://bioinfo.ut.ee/primer3-0.4.0/primer3/) and were selected wherever possible to amplify sequences that spanned at least one intron. Primer sequences were analyzed for *T*_*m*_ (melting temperature), secondary structure and primer-dimer formation with NetPrimer analysis software (http://www.premierbiosoft.com/netprimer). PCR assays were performed using the CFX cycler (Bio-Rad) and all samples were run in duplicate. All mRNAs amplified with high efficiency and linearity during real-time PCR. Mean amplification efficiencies, as determined by plotting cycle threshold as a function of initial cDNA quantity, were in the range of 1.90–2.00. Results obtained from the qPCR experiments were, therefore, quantified using the comparative threshold cycle (C_T_) method (ΔΔC_T_) for relative quantitation of gene expression, with a minor correction for amplification efficiency (Pfaffl, 2001). The ONH tissue extracts prepared via biopsy punch contain relatively low, but variable, amounts of central retina. Consequently, investigation into whether certain mRNAs, for example LDH-A, are upregulated in ONH astrocytes following induction of ocular hypertension, is complicated by the fact that LDH-A, for example, is abundantly expressed in healthy photoreceptors and bipolar cells. To eliminate the influence of endogenous expression of mRNAs within the retina, all pPCR values were normalized to rhodopsin (which acts as a control for retinal content) and then expressed relative to controls. Statistical analysis was carried out by Student's unpaired *t*-test. The null hypothesis tested was that normalized C_*T*_ differences of target genes would be the same in control and treated ONH samples.

**Table 2:** Primer sequences for mRNAs amplified by real-time RT-PCR.

**mRNA**	**Primer sequences**	**Product size (bp)**	**Annealing temperature**	**Accession number**
Rhodopsin	5′-CTCCATCTACAACCCAATCATC-3′	187	63°C	NM_033441
	5′-ACTCCTACAGTCAGCCACAGTC-3′			
GCLM	5′-ATCTTGCCTCCTGCTGTGT-3′	95	60°C	NM_017305
	5′-CAGTTCTTTTGGGTCATTGTG-3′			
LDH-A	5′-GCACTAAGCGGTCCCAAAAG-3′	126	63°C	NM_017025
	5′-ACAGCACCAACCCCAACAAC-3′			
NQO1	5′-GGCTCTGAAGAAGAAAGGATGG-3′	95	62°C	NM_017000
	5′-GCTCCCCTGTGATGTCGTT-3′			
NOX-2	5′-CACCCTTTCACCCTGACCTCT-3′	215	63°C	NM_023965
	5′-GCTCCCACTAACATCACCACCT-3′			
Nrf2	5′-CGAAAAGGAGAGACAAGAGCAA-3′	160	62°C	NM_031789
	5′-GTGGGCAACCTGGGAGTAG-3′			
Prdx6	5′-AAACTAAAACTGTCCATCCTCTACC-3′	143	59°C	NM_053576
	5′-ACCATCACACTCTCTCCCTTCT-3′			
Transferrin receptor	5′-GTTTCCGCCATCTCAGTCATC-3′	243	61°C	NM_022712
	5′-CGGTCTGGTTCCTCATAGCC-3′			

### Western immunoblotting

Electrophoresis/Western blotting was performed as previously described (Chidlow et al., 2010). In brief, ONH protein samples were prepared from TRI-reagent extracts as per the manufacturer's protocol. Extracted proteins were solubilized in homogenization buffer containing 1% SDS, diluted with an equal volume of sample buffer, and boiled for 3 min. Electrophoresis was performed using non-denaturing 10% polyacrylamide gels. After separation, proteins were transferred to polyvinylidine fluoride membranes for immunoprobing. Blocking of membranes was carried out in a solution of tris-buffered saline containing 0.1% (v/v) Tween-20 and 5% (w/v) non-fat dried skimmed milk. Membranes were then incubated consecutively with the appropriate primary antibody (Table 1), biotinylated secondary antibody and streptavidin-peroxidase conjugate; inter-step washes were carried out in tris-buffered saline (pH 7.4) containing 0.1% (v/v) Tween-20. Color development was achieved using 3-amino-9-ethylcarbazole. Images were captured and analyzed for densitometry using the program, Adobe PhotoShop CS2. Densitometry values were normalized for actin. Statistical analysis was carried out by Student's unpaired *t-*test (control group vs. OHT group). The null hypothesis tested was that densitometry measurements for target proteins (normalized for actin) would be the same in control and OHT samples.

## Results

### Ocular hypertension causes a variable degree of axonal transport disruption at the optic nerve head

Previous studies, including our own, have shown that laser photocoagulation of the limbal tissues leads to a substantial and immediate elevation of IOP in the treated eye with the peak value occurring within the first 24 h (see Vidal-Sanz et al., 2011). Of the 16 rats injected with pimonidazole and detailed in Table 3, the mean baseline IOP was 12.1 ± 2.9 mm Hg (mean ± *SD*) and the mean IOP at time of death (24 h post-laser) was 36.7 ± 6.0 mm Hg. A similar magnitude of OHT was attained with all of the other rats analyzed in this study (data not shown).

**Table 3:** Characteristics of pimonidazole-injected rats.

**Rat**	**IOP (baseline)**	**IOP (at 1 d)**	**Axonal transport disruption grade**	**Pimonidazole labeling (ONH)**	**HO-1 labeling (ONH)**
1	12	40	Medium	No	Yes
2	10	36	Low	No	Yes
3	14	38	High	Yes	Yes
4	16	39	High	Yes	Yes
5	16	47	High	Yes	Yes
6	15	44	Medium	Yes	Yes
7	8	35	Medium	Yes	Yes
8	9	34	Low	No	Yes
9	11	25	Low	No	Yes
10	9	31	Medium	No	Yes
11	12	27	Medium	Yes	Yes
12	14	44	High	Yes	Yes
13	16	38	High	Yes	Yes
14	14	38	Medium	Yes	Yes
15	8	32	Medium	Yes	Yes
16	10	39	Medium	Yes	Yes

Following 24 h of OHT, a proportion of RGC fibers passing through the ONH feature disrupted orthograde axonal transport. This can be visualized using the neural tracer cholera toxin B subunit, or by immunolabeling with antibodies directed against amyloid precursor protein (APP), or interleukin-6 (Chidlow et al., 2011b, 2012). In the current study, we used both APP and interleukin-6 to visualize compromised axons. There can be marked inter-animal variability in the extent of axonal transport disruption after induction of OHT. Using a semi-quantitative scoring system, ONH sections can be categorized as low, medium or high (see Figure 1 for representative images). In general, rats with higher IOPs display greater axonal transport injury (Chidlow et al., 2011b). Categorization of the rats detailed in Table 3 highlights this correlation: mean IOP for animals having their level of damage categorized as low = 31.7 ± 5.9 mm Hg; medium = 35.8 ± 5.5 mm Hg; high = 41.2 ± 4.1 mm Hg. Nevertheless, on an individual basis, IOP (as determined by rebound tonometry) is not a reliable predictor of pathology. Moreover, due to the sectorial nature of RGC injury in glaucoma, variability in the amount of axonal transport disruption is even evident when comparing different levels within the same ONH. It is important to consider these issues when assessing whether OHT is associated with hypoxia.

### Ocular hypertension leads to selective hypoxia at the optic nerve head

**Figure 2:**
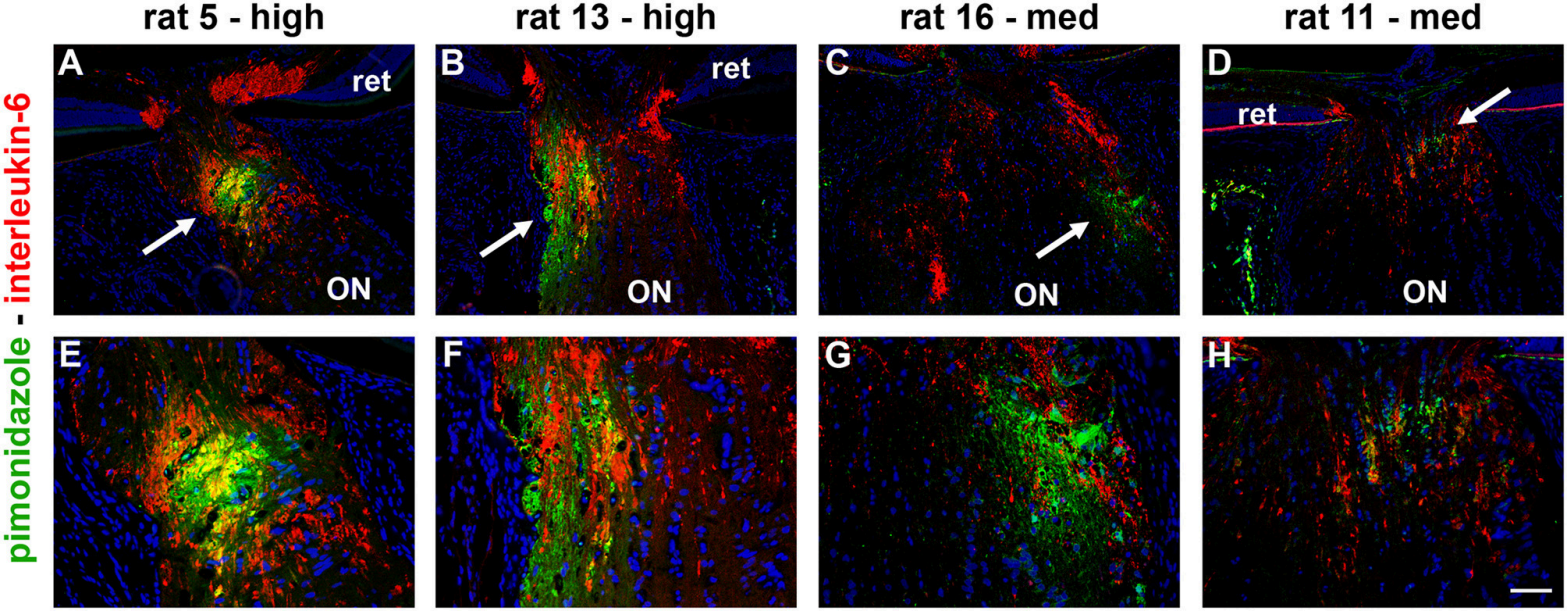
Localization of hypoxia within the optic nerve head (ONH) at 24 h following induction of ocular hypertension. **(A–H)** Double labeling immunofluorescence of interleukin-6 (red), to demarcate those axons with disrupted axonal transport, with pimonidazole (green), to highlight hypoxic regions (arrows), in four representative animals. **(A–D)** are low magnification images, while **(E–H)** show the same sections at higher magnification. In the examples presented, the rats with high axonal transport disruption feature larger hypoxic regions than the rats with medium axonal transport disruption. The position of the hypoxic region can be seen to vary markedly between animals. ON, optic nerve; ret, retina. Scale bars: **(A–D)** = 100 μm; **(E–H)** = 50 μm.

**Figure 3:**
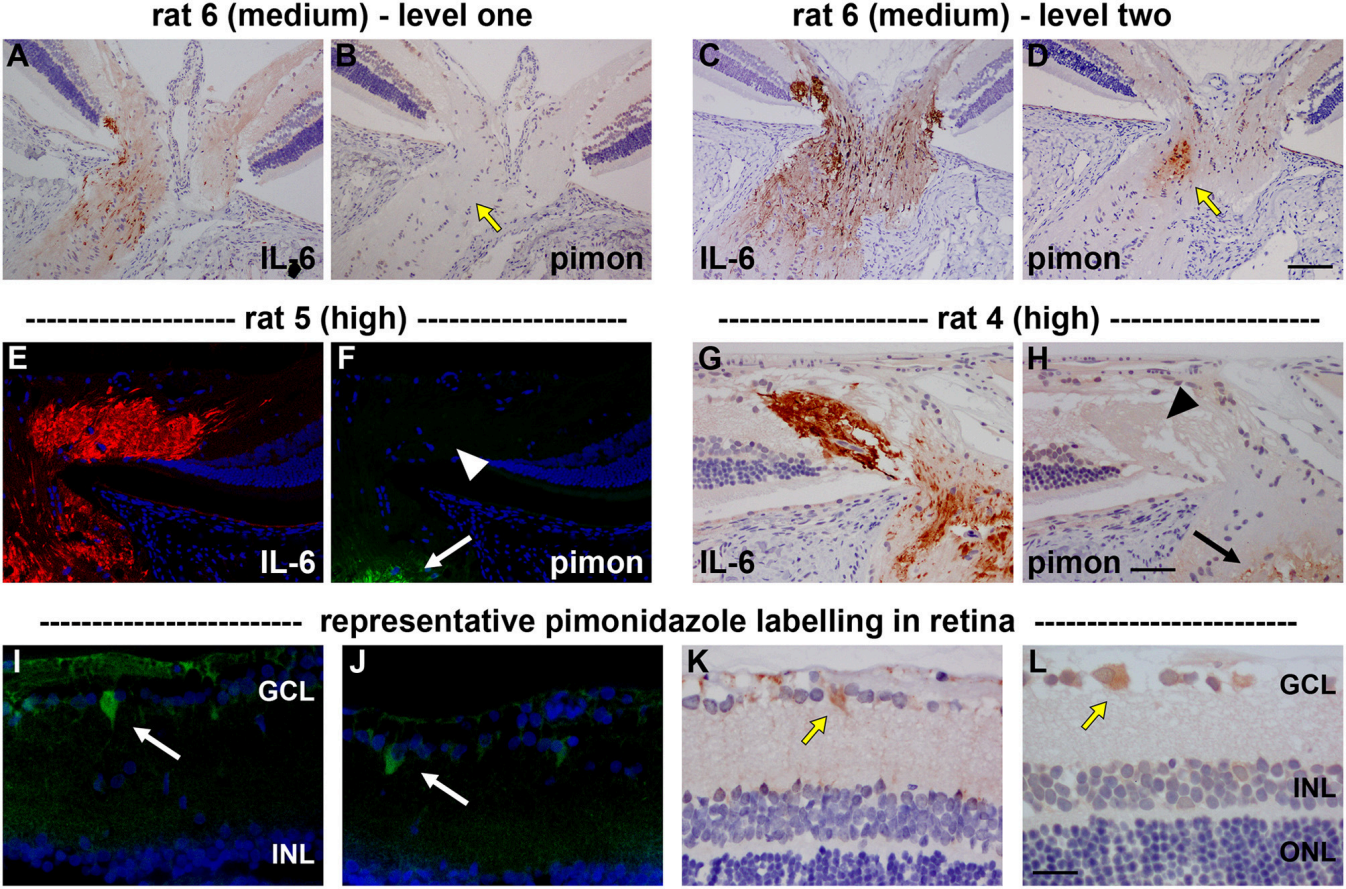
Localization of hypoxia within the optic nerve head (ONH) and retina at 24 h following induction of ocular hypertension. **(A–D)** Hypoxic regions can be very focal, as shown by two images of pimonidazole (pimon) staining in the same rat ONH **(B,D)**, each with an accompanying image of interleukin-6 (IL-6) to demarcate the number of axons with disrupted axonal transport **(A,C)**: hypoxia is not detectable in (**B**, yellow arrow), but a small hypoxic region is apparent in (**D**; yellow arrow). It is notable that positive staining for pimonidazole is only observed in the portion of the ONH that displays greater axonal transport disruption (**C** vs. **A**). **(E–H)** Pimonidazole staining is not evident in the prelaminar ONH (**F**,**H**, white and black arrowheads), even in animals with prominent axonal transport disruption at this location **(E,G)** and featuring hypoxic regions within the laminar ONH (**F,H**, white and black arrows). **(I–L)** In the retina, very few cells stain positively for pimonidazole. In animals with high axonal transport disruption, occassional cells in the GCL are observed (white and yellow arrows). GCL, ganglion cell layer; INL, inner nuclear layer; ONL, outer nuclear layer. Scale bars: **(A–D)** = 100 μm; **(E–H)** = 50 μm; **(I–L)** = 25 μm.

**Figure 4:**
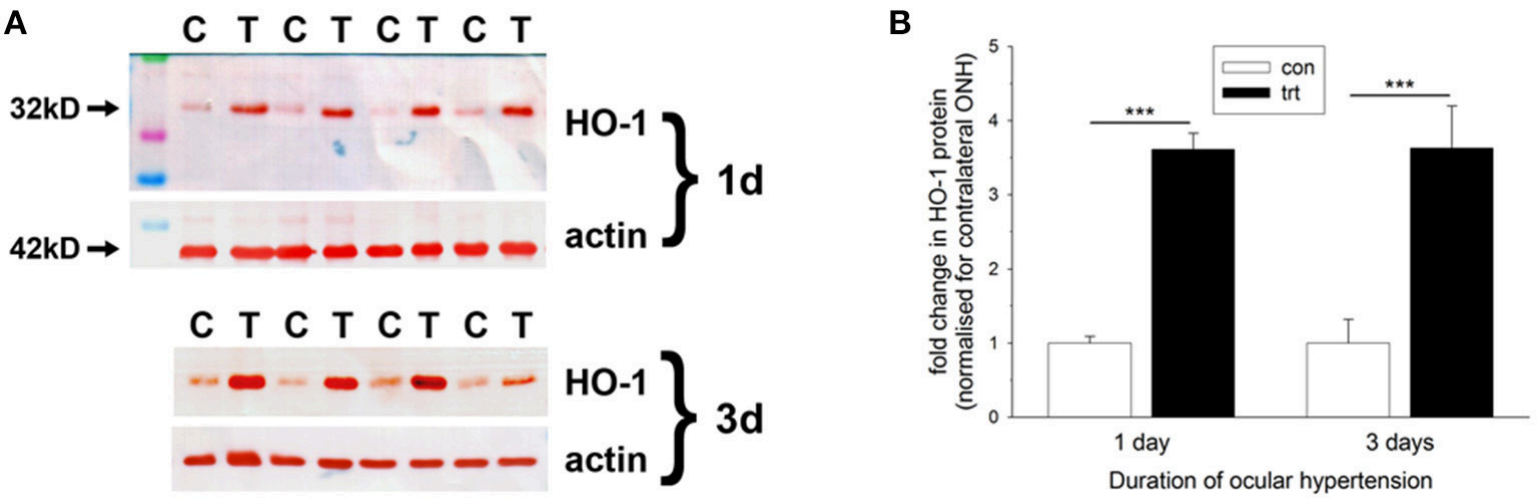
Evaluation of heme oxygenase-1 (HO-1) expression in optic nerve head (ONH) extracts at 1 and 3 d after induction of ocular hypertension, as determined by Western immunoblotting. **(A)** Representative immunoblots from four animals at each time point are shown (C, control ONH; T, treated ONH). Single bands of the expected molecular weight are apparent. β-actin immunoblots for the same samples are also shown, as gel-loading controls. **(B)** Quantification of HO-1 protein data. Values (represented as mean ± SEM, where *n* = 12–14) are normalized for actin and expressed relative to controls. ^***^P < 0.001, by Student's unpaired *t-*test.

To detect any cellular hypoxia, pimonidazole was infused systemically 3 h prior to rats being humanely killed. As detailed in the Materials and Methods Section, pimonidazole forms stable covalent adducts with cells that have an oxygen partial pressure of <10 mmHg (Arteel et al., 1995), which can then be localized immunohistochemically in tissue sections. No pimonidazole labeling was evident in the optic nerve, ONH or retina of any of the untreated eyes (data not shown). Of the 16 OHT eyes, positive staining was detected within the ONH of 11 of them (Table 3). The 11 eyes positive for pimonidazole had a mean IOP of 38.3 ± 5.7 mm Hg, while the 5 negative eyes had a mean IOP of 33.2 ± 5.6 mm Hg, a difference that did not reach statistical significance (*P* = 0.11 by Student's unpaired *t-*test). Subdivision of the eyes by axonal transport disruption category revealed the following: 0/3 graded low for axonal transport disruption were positive for pimonidazole; 6/8 graded medium were positive; 5/5 graded high were positive (Table 3). A number of points are worth making regarding pimonidazole staining in OHT eyes: (1) no two animals were alike as regards the area or position of hypoxia; (2) pimonidazole always labeled in areas of the ONH featuring injured axons; (3) the greater the abundance of axonal transport disruption, the greater the likelihood of a large hypoxic region; (4) pimonidazole staining was observed in glial cells as well as axons; (5) hypoxic regions were sometimes very focal and not evident in sections taken deeper within the same ONH; (6) disrupted axonal transport was frequently encountered without any pimonidazole labeling; (7) no pimonidazole staining was observed in the prelaminar ONH, even in animals with prominent axonal transport disruption at this location; (8) no pimonidazole staining was observed in the portion of the optic nerve distal to the ONH. Representative images of pimonidazole staining in OHT eyes are provided in Figures 2–**4**. The images shown illustrate the points made above. Overall, the experiments with pimonidazole show that oxygen availability to regions within the ONH is reduced to hypoxic levels in some OHT eyes. It is highly likely that oxygen availability is reduced to some degree throughout the ONH, just not to the extent needed to produce covalent adducts of pimonidazole.

With regard to the retina, pimonidazole stained occassional RGCs in OHT eyes, typically in specimens categorized as high for axonal transport disruption. No unambiguous positive staining was observed in other classes of cells within the retina (Figure 3).

### Ocular hypertension induces heme oxygenase-1 expression in optic nerve head astrocytes

Low tissue oxygen availability can lead to a multitude of gene transcription changes mediated primarily via stabilization of two key transcription factors: hypoxia-inducible factor-1a (HIF-1) and nuclear factor erythroid 2-related factor 2 (Nrf2). HIF-1α and Nrf2, respectively, bind to and activate genes that possess the hypoxia response element and the antioxidant response element. HRE target genes are classically involved in oxygen homeostasis and anaerobic energy metabolism (Guillemin and Krasnow, 1997), while ARE target genes are characteristically antioxidant and detoxification enzymes (Wasserman and Fahl, 1997). Heme oxygenase-1 (HO-1) is a gene target of both Nrf2 and HIF-1. If low oxygen availability and increased production of ROS are involved in axonal transport disruption following induction of OHT, then HO-1 would be expected to be induced by neighboring glial cells, as occurs in the brain (Schipper et al., 2009).

**Figure 5:**
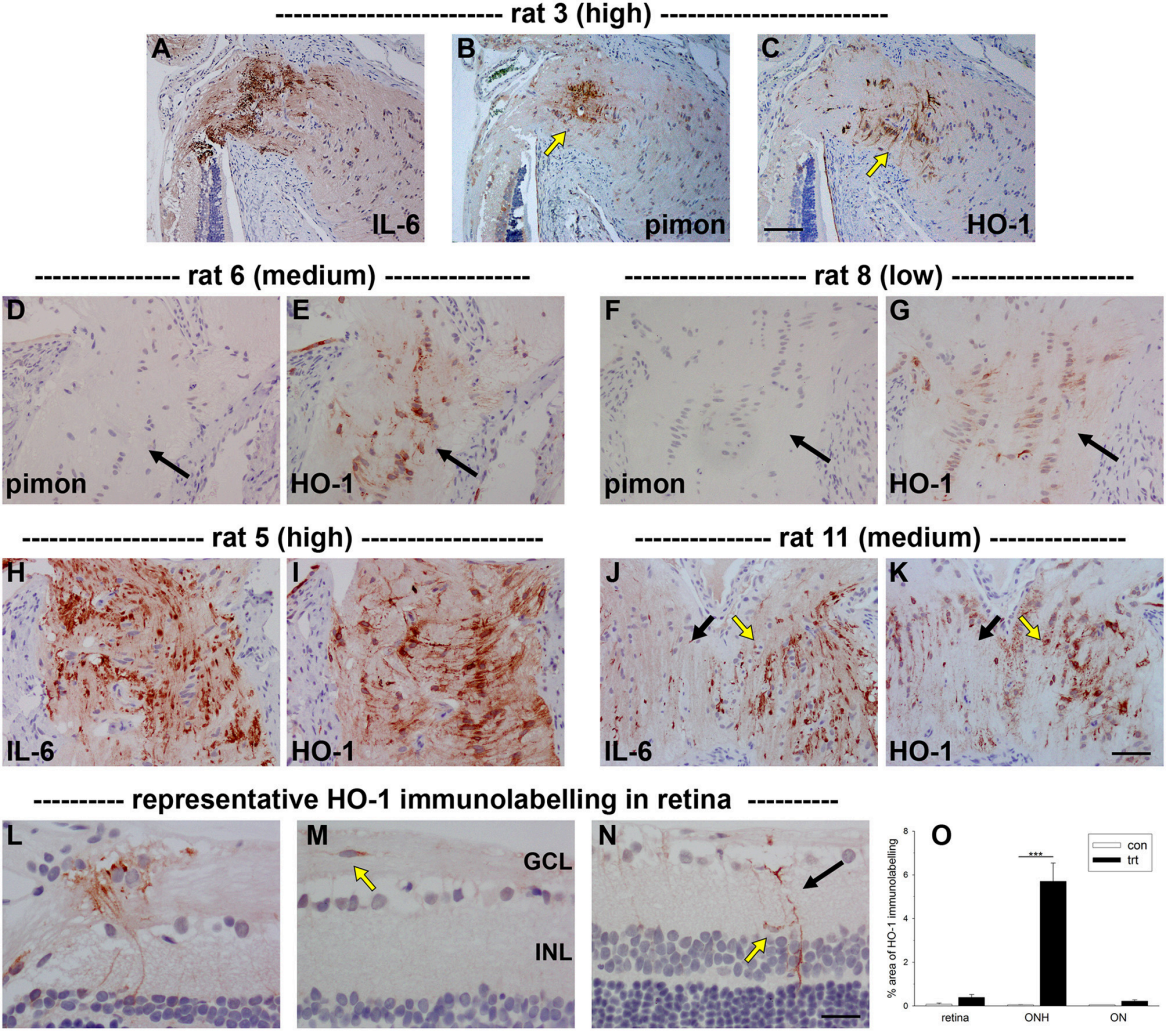
Localization of heme oxygenase-1 (HO-1) immunolabeling within the optic nerve head (ONH) and retina at 24 h following induction of ocular hypertension. **(A–C)** Images of axonal transport disruption as delineated by interleukin-6 (IL-6), pimonidazole (pimon) staining to demarcate any hypoxia, and HO-1, all within the same plane of the ONH of rat 3. It can be seen that the distribution of HO-1 overlaps closely with those of axonal injury and hypoxia, but is somewhat more extensive than the latter (arrows). **(D–G)** Of note, HO-1 is upregulated by ONH glial cells in ocular hypertensive rats that do not feature an overt hypoxic region. **(H–K)** HO-1 is upregulated more robustly in rats that feature abundant axonal transport disruption. The distribution of HO-1 immunolabeling closely parallels that of IL-6 (see **J,K**: black arrows, low IL-6/HO-1 expression; yellow arrows, high IL-6/HO-1 expression). **(L–N)** In the retina, HO-1 immunolabeling is sparse at 24 h. Macroglial cells in the prelaminar ONH are often HO-1-positive **(L)**. HO-1-labeled microglia located within the nerve fiber layer can sometimes also be observed **(M)**. In the mid-central retina, Müller cell processes (black arrow) and microglia present within the inner plexiform layer (yellow arrow) occassionally express HO-1 **(N)**. GCL, ganglion cell layer; INL, inner nuclear layer. Scale bars: **(A–C)** = 100 μm; **(D–K)** = 50 μm; **(H–J)** = 25 μm. **(O)** Quantification of HO-1 immunolabeling in the retina, optic nerve head (ONH) and myelinated optic nerve (ON). Values represent mean ± SEM, where *n* = 24. ^***^P < 0.001, by Student's unpaired *t-*test.

Initially, we investigated HO-1 protein expression in dissected ONH samples (see Section Materials and Methods) from rats subjected to 1 and 3 d of OHT. The data showed a robust, statistically significant (*P* < 0.001) upregulation of HO-1 in treated ONH extracts at both time points (Figure 4). Next, we performed immunolabeling of HO-1 in tissue sections. No consistent patterns of HO-1 immunolabeling were evident in the optic nerve, ONH or retina of any of the contralateral, untreated eyes (data not shown). After 1 d of OHT, HO-1 immunolabeling was upregulated within ONH astrocytes (Figure 5, Supplementary Figure 1, Table 3). All rats analyzed, including the 16 pimonidazole-injected rats, displayed positive staining by ONH astrocytes (Figure 5). In the pimonidazole-injected rats, HO-1 was upregulated in the hypoxic region and immediate penumbra (Figures 5A–C). HO-1 expression was also observed in ONH sections of pimonidazole rats that did not feature an overt hypoxic region, including ONHs categorized as low for axonal transport disruption (Figures 5D–G). The extent of HO-1 expression closely matched the abundance of axonal transport disruption (Figures 5H–K). At 1 d after induction of OHT, HO-1 was largely restricted to the ONH. Quantification of the area of HO-1 labeling in images taken from the central retina, ONH and myelinated optic nerve revealed a dramatic increase within the ONH, but only subtle changes in the retina and optic nerve (Figure 5O). In the retina, HO-1 was most commonly associated with astrocytes/Müller cell end feet in the prelaminar ONH. Occasional astrocytes, Müller cells, and microglia were HO-1-positive within the body of the retina (Figures 5L–N). After 3 d of OHT, the distribution of HO-1 immunolabeling was more widespread, encompassing retinal and ONH microglia, as well as astrocytes, patches of Müller cells and occassional RGCs (Supplementary Figure 1).

### Ocular hypertension upregulates lactate dehydrogenase-A expression in the optic nerve head

**Figure 6:**
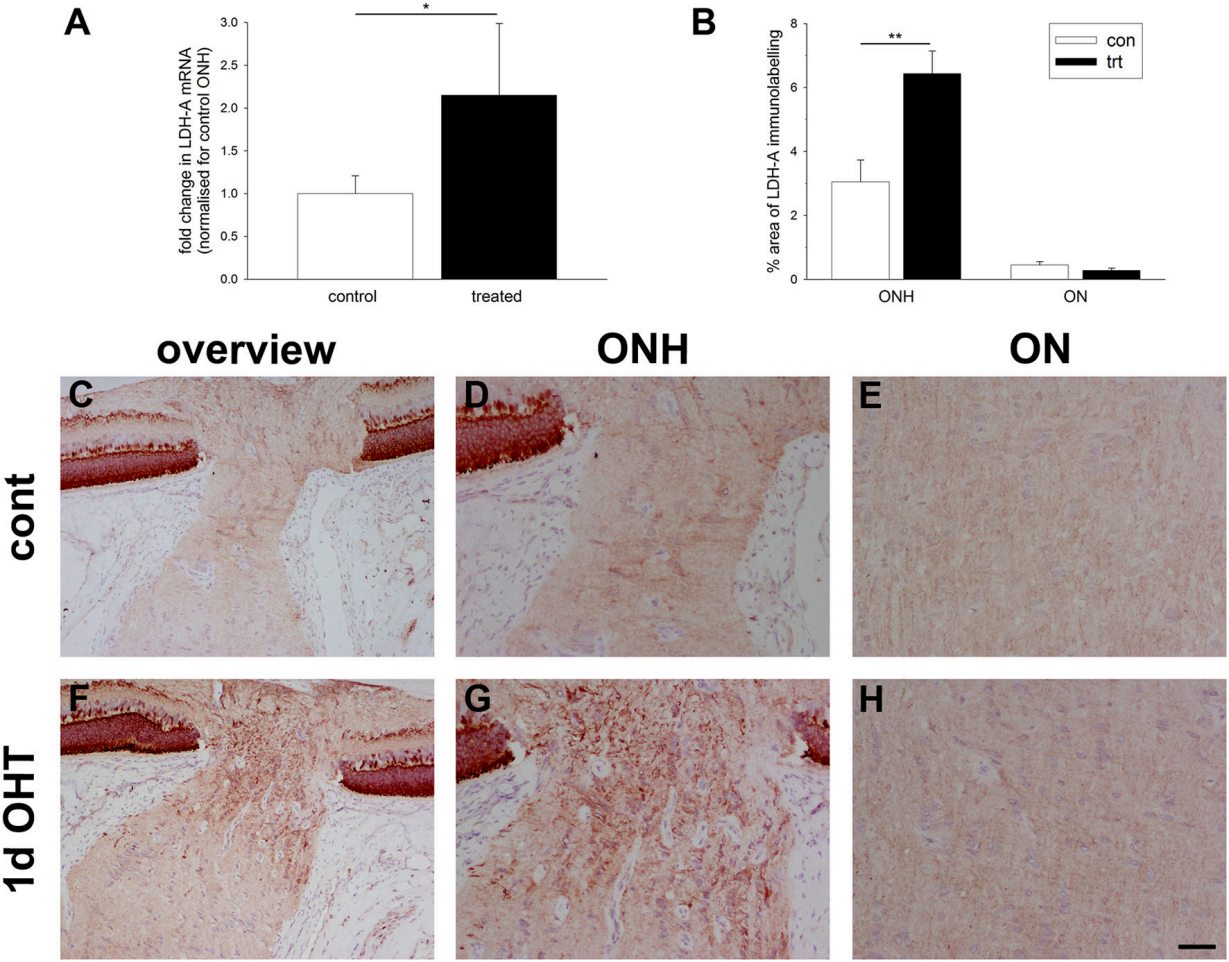
Evaluation of lactate dehydrogenase-A (LDH-A) expression in the optic nerve head (ONH) at 1 d after induction of ocular hypertension. **(A)** Level of LDH-A mRNA in ONH samples from OHT eyes, as measured by qPCR. Data are normalized for rhodopsin and expressed relative to controls. Values represent mean ± SEM, where *n* = 14. ^*^P < 0.05, by Student's unpaired *t-*test. **(B)** Quantification of LDH-A immunolabeling in the ONH and myelinated optic nerve (ON). Values represent mean ± SEM, where *n* = 14. ^**^P < 0.01, by Student's unpaired *t*-test. **(C–H)** Representative images of LDH-A immunolabeling within the ONH and proximal portion of the myelinated optic nerve (ON) of a control eye and an eye analyzed at 24 h after induction of ocular hypertension. **(C**,**F)** are low magnification images, while **(D**,**E**,**G**,**H)** show the same sections at higher magnification. In control rats, LDH-A is weakly expressed throughout retinal ganglion cell axons and by glial cells of the ONH. In treated animals, LDH-A expression is markedly upregulated within the ONH, but not ON. Scale bar: **(C,F)** = 100 μm; **(D,E,G,H)** = 50 μm.

Under conditions of reduced oxygen availability, metabolism of glucose to lactate via anaerobic glycolysis becomes the principal route by which cells generate ATP. Glycolytic genes have been repeatedly identified as inducible by hypoxia (Hu et al., 2003). To explore whether there is an alteration in glycolytic machinery within the ONH at 1 d after induction of OHT, we investigated expression of lactate dehydrogenase-A (LDH-A). qPCR analysis of dissected ONH samples revealed a statistically significant two-fold upregulation of LDH-A mRNA in the treated eye compared to the contralateral eye (*P* < 0.05; Figure 6A). Immunolabeling for LDH-A in control eyes revealed abundant expression within photoreceptors and bipolar cells in the retina, and weak labeling of axons and glial cells in the optic nerve (Figures 6C–E), in agreement with previous work (Casson et al., 2016). Following induction of elevated IOP, stronger expression of LDH-A was observed within the ONH, but never more distally in the optic nerve (Figures 6F–H). The upregulated expression within the ONH encompassed glial cells and possibly axons. Quantification of the area of LDH-A labeling in images taken from the ONH and myelinated optic nerve confirmed the observed increase within the ONH (*P* < 0.01), but not the optic nerve (Figure 6B).

### Ocular hypertension selectively affects iron-regulating proteins in the optic nerve head

**Figure 7:**
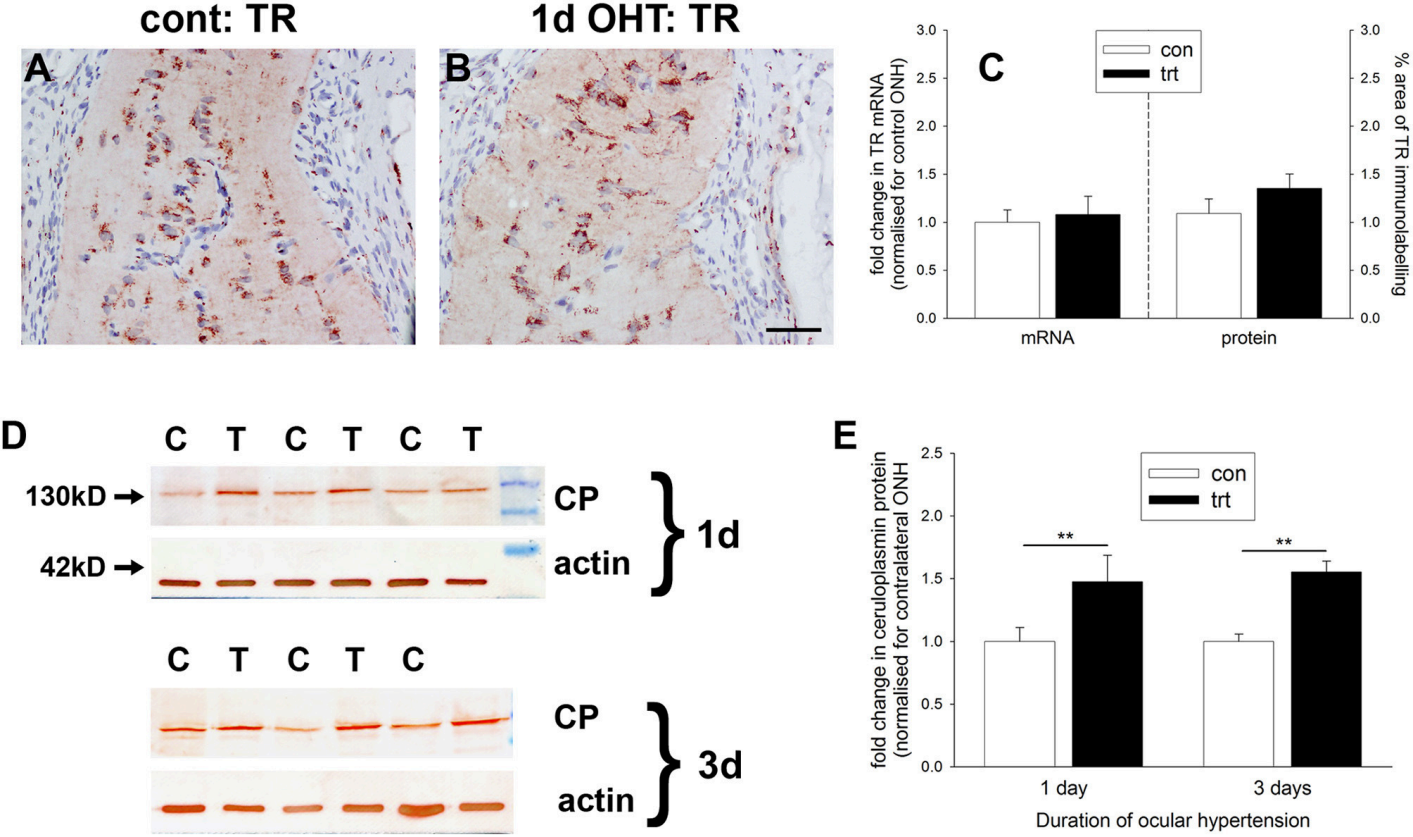
Evaluation of iron-regulating proteins transferrin receptor and ceruloplasmin in the optic nerve head (ONH) at 1 and 3 d after induction of ocular hypertension **(A,B)** Representative images of transferrin receptor (TR) immunolabeling within the ONH of a control eye and an eye analyzed at 24 h after induction of ocular hypertension. Scale bar: 50 μm. **(C)** Quantification of transferrin receptor mRNA expression in ONH extracts, as measured by qPCR, and transferrin receptor protein expression in tissue sections of the ONH. All values represent mean ± SEM, where *n* = 14. pPCR data are normalized for rhodopsin and expressed relative to controls. **(D)** Representative immunoblots from three animals at each time point are shown (C, control ONH; T, treated ONH). Single bands of the expected molecular weight are apparent. β-actin immunoblots for the same samples are also shown, as gel-loading controls. **(E)** Quantification of ceruloplasmin protein data. Values (represented as mean ± SEM, where *n* = 12–14) are normalized for actin and expressed relative to controls. ^**^P < 0.01, by Student's unpaired *t-*test.

Iron is crucial to the transport of molecular oxygen to tissues and its depletion has the capacity to invoke or worsen hypoxia (Chepelev and Willmore, 2011). A number of iron homeostasis genes, including transferrin, transferrin receptor, ceruloplasmin, and HO-1, are transcriptionally upregulated by hypoxia in a HIF-dependent manner (Chepelev and Willmore, 2011), while ceruloplasmin has additionally been credited with possessing antioxidant properties. To explore whether there is an alteration in iron-regulating proteins within the ONH during OHT, we investigated expression of transferrin receptor and ceruloplasmin. qPCR analysis of dissected ONH samples revealed no alteration in transferrin receptor mRNA in the treated eye compared to the contralateral eye (*P* = 0.74; Figure 7C). Immunolabeling for transferrin receptor in controls showed punctate labeling of glial cells in the ONH and optic nerve (Figure 7A). In agreement with earlier work (Moos, 1996), capillary endothelial cells and columns of oligodendrocytes were clearly positive for transferrin receptor (Supplementary Figure 2). Following induction of elevated IOP, no discernible alteration to the distribution of transferrin receptor was evident (Figure 7B). Quantification of the area of immunolabeling in images taken from the ONH confirmed this observation (*P* = 0.22; Figure 7C).

In contrast to transferrin receptor, ceruloplasmin protein levels in dissected ONH samples from rats subjected to both 1 or 3 d of OHT were found to be significantly higher than in controls (*P* < 0.01 for both) when analyzed by Western blotting (Figures 7D,E). Ceruloplasmin displays a pattern of immunolabeling within the control (Supplementary Figure 2) and treated (data not shown) ONH that is characteristic of astrocytes, suggesting that the increase in protein expression following ocular hypertension derives from upregulation by this cell type.

### Ocular hypertension induces AP-1 expression in the optic nerve head

**Figure 8:**
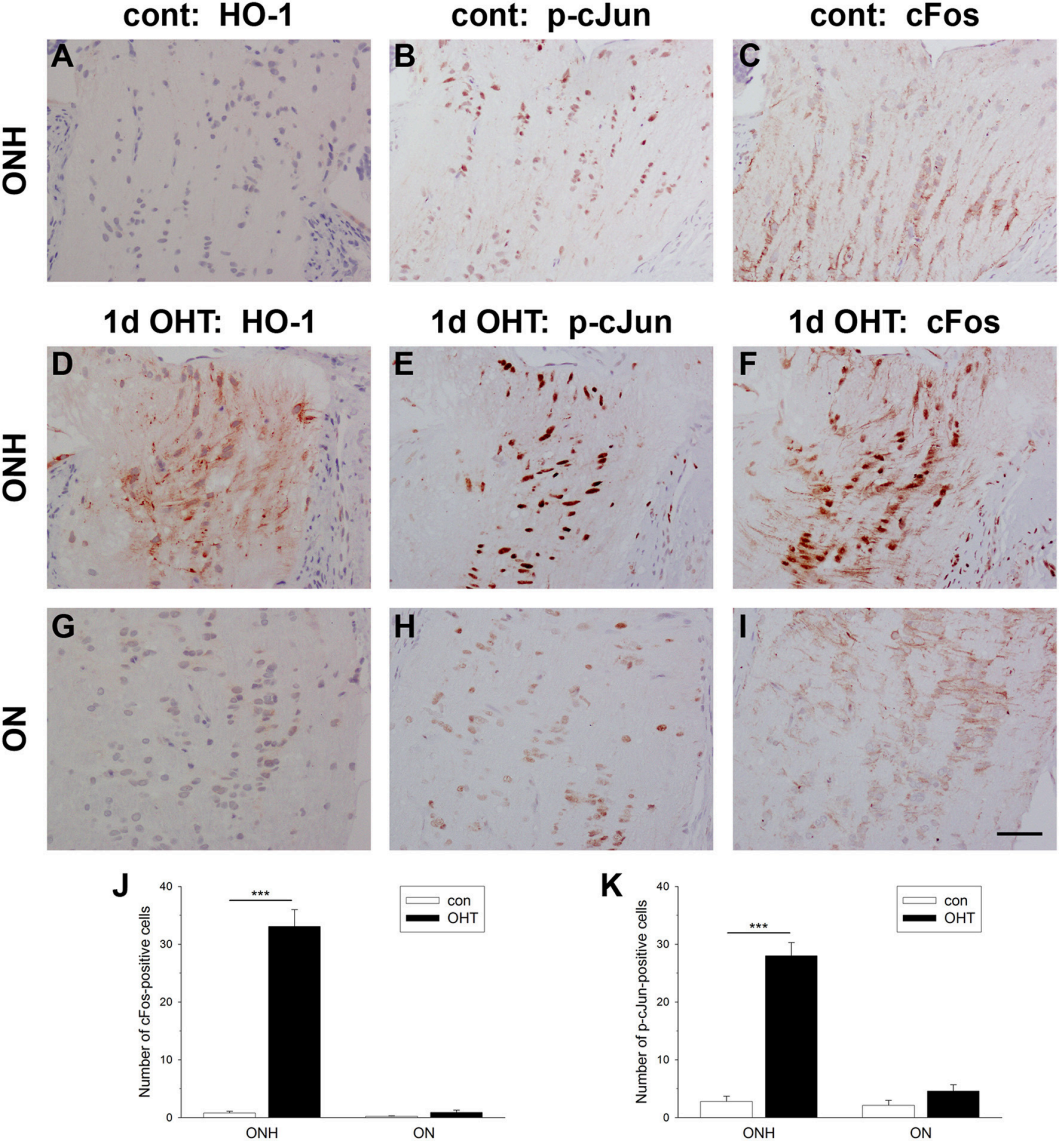
Evaluation of cFos and p-cJun expression in the optic nerve head (ONH) at 1 d after induction of ocular hypertension (OHT). **(A–C)** Representative images of heme oxygenase-1 (HO-1), cFos, and p-cJun immunolabeling within a control ONH. **(D–I)** Representative images of HO-1, cFos, and p-cJun immunolabeling within the ONH and proximal portion of the optic nerve (ON) of a rat analyzed at 24 h after induction of ocular hypertension. In the control ONH, few cell nuclei label positively for cFos or p-cJun. In contrast, at 24 h after induction of OHT, numerous nuclei within the ONH, but not the ON, are cFos- and p-cJun-positive. Heme oxygenase-1 (HO-1) immunolabeling from the same rat is shown for comparative purposes. Scale bar: 50 μm. **(J,K)** Quantification of cFos and p-cJun immunolabeling in the ONH and ON. Values represent mean ± SEM, where *n* = 24. ^***^P < 0.001, by Student's unpaired *t-*test.

Activating protein-1 (AP-1), a pleiotropic transcription factor and member of the leucine zipper family, is a dimer composed of JUN/FOS or JUN/JUN subunits. AP-1 activation by transient and prolonged hypoxia is a well-described phenomenon (Cummins and Taylor, 2005). To explore whether AP-1 activation at the ONH corresponds temporally with axonal transport disruption, we quantified the number of nuclei expressing cFos and the active, phosphorylated form of cJun (p-cJun) at 1 d after induction of OHT in images taken from the ONH and myelinated optic nerve. In control eyes, very few cells in either the ONH or proximal optic nerve displayed nuclear expression of cFos or p-cJun (Figures 8B,C,J,K). In treated eyes, however, there was a striking increase in expression of both cFos (*P* < 0.001) and p-cJun (*P* < 0.001) at the ONH (Figures 8E,F,J,K). The inductions of cFos (*P* = 0.11) and p-cJun (*P* = 0.10) did not extend spatially into the optic nerve (Figures 8H–K). The spatial extent of cFos and p-cJun upregulations were comparable to that of HO-1 (Figures 8A,D,G). Moreover, double labeling with the glial marker S100 revealed that expression of both cFos and p-cJun was restricted to astrocytes (Supplementary Figure 3).

### Ocular hypertension induces only modest alterations to Nrf2 and Nrf2-driven antioxidant enzymes in the optic nerve head

**Figure 9:**
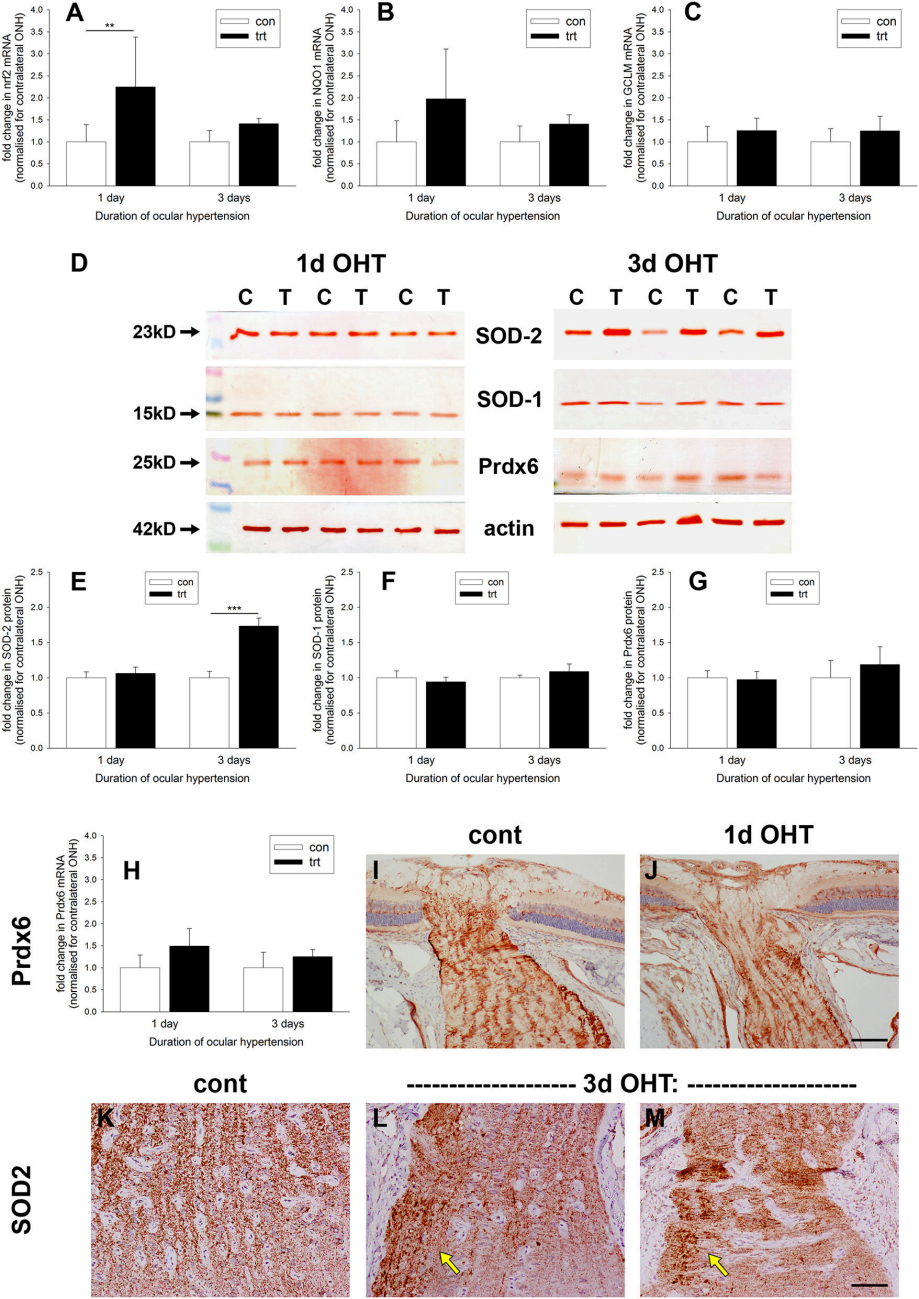
Evaluation of antioxidant defenses in the optic nerve head (ONH) after induction of ocular hypertension (OHT). **(A–C)** Quantification of mRNAs encoding the antioxidant response element genes Nrf2, GCLM, and NQO1 in ONH extracts, as measured by qPCR. Data are normalized for rhodopsin and expressed relative to controls. Values represent mean ± SEM, where *n* = 12–14. ^**^P < 0.01, by Student's unpaired *t-*test. **(D–G)** Expression of the antioxidant enzymes SOD-1, SOD-2, and Prdx6 in optic nerve head (ONH) extracts at 1 and 3 d after induction of ocular hypertension, as determined by Western immunoblotting. Representative immunoblots from three animals at each time point are shown (C, control ONH; T, treated ONH). Single bands of the expected molecular weight are apparent. β-actin immunoblots for the same samples are also shown, as gel-loading controls. Values (represented as mean ± SEM, where *n* = 12–14) are normalized for actin and expressed relative to controls. ^***^P < 0.001, by Student's unpaired *t*-test. **(H)** Quantification of Prdx6 mRNA expression in ONH extracts, as measured by qPCR. All values represent mean ± SEM, where *n* = 14. Data are normalized for rhodopsin and expressed relative to controls. **(I,J)** Representative images of Prdx6 immunolabeling in ONH tissue sections from control and OHT eyes. Prdx6 is associated with astrocytes, but appears unchanged following OHT. **(K–M)** Representative images of SOD-2 immunolabeling in ONH tissue sections from control and eyes subjected to 3 d of OHT. In controls optic nerves, SOD-2 expression is conspicuous within axon bundles. Following 3 d of OHT, injured axon bundles display robust, punctate SOD-2 immunolabeling (arrows). Scale bar: **(I,J)** = 100 μm; **(K–M)** = 50 μm.

Oxidative stress, brought about by excessive production of ROS, is implicated in the pathogenesis of RGC loss during glaucoma (Tezel, 2006). Upon redox perturbation, the transcription factor Nrf2 controls the inducible expression of a multitude of genes involved in protection against oxidative stress (Tebay et al., 2015). We investigated expression of Nrf2 itself and some Nrf2-responsive genes at the ONH during OHT. qPCR data of dissected ONH samples revealed an increase in Nrf2 mRNA in the treated vs. the contralateral eye of approximately two-fold (*P* < 0.01) after 1 d of OHT (Figure 9A); however, by 3 d Nrf2 mRNA was no longer significantly elevated (*P* = 0.28). Examination of the levels of two key Nrf2 target genes, NQO1 and GCLM, failed to show any robust increases in expression at either time point (Figures 9B,C), although the former transcript almost reached significance at the 1 d time point (*P* = 0.06).

Western blotting data (Figures 9D–G) of dissected ONH samples probed with antibodies directed against the cytoplasmic (SOD-1) and mitochondrial (SOD-2) isoforms of superoxide dismutase and the peroxide scavenging enzyme peroxiredoxin-6 (Prdx6) revealed no alterations in any of the three enzymes after 1 d of OHT (*P* = 0.50, *P* = 0.51, *P* = 0.80, respectively). By 3 d of OHT, SOD-1, and Prdx6 remained unchanged relative to normotensive eyes (*P* = 0.24, *P* = 0.30, respectively). Interestingly, SOD-2 was consistently elevated at this later time point (*P* < 0.001). We augmented these Western blotting results by performing qPCR on ONH extracts and immunolabeling for Prdx6 and SOD-2 in ONH tissue sections. The rationale for these experiments was simply that Prdx6 is the peroxiredoxin isoform expressed exclusively by astrocytes within the ONH (Chidlow et al., 2016) and hence is of obvious interest, whilst SOD-2 displayed an upregulated profile at the 3 d time point and the cellular origin of this increase is of relevance. As regards Prdx6, neither qPCR nor immunohistochemistry suggested an upregulation of this enzyme during the early stages of OHT (Figures 9H–J). In contrast, SOD-2 is present in all cells, but within the ONH is predominantly visible, as expected, within the unmyelinated axons (Figure 9K). After 3 d of OHT, more intense, punctate SOD-2 labeling of axons was evident in zones of axonal injury (Figures 9L,M); thus, it appears plausible that axons rather than astrocytes represent the source of the additional SOD-2 that was detected by Western blotting.

### Ocular hypertension increases generation of superoxide in the retina and optic nerve head

Superoxide represents the primary ROS generated during oxidative stress. Analysis of superoxide formation, accordingly, represents a useful tool for identifying the likely sites of any oxidative stress. Notwithstanding the mitochondrial respiratory chain, the major cellular source of superoxide in disease scenario is thought to be the dedicated superoxide-generating enzyme NADPH oxidase (NOX), in particular the NOX-2 isoform (Bedard and Krause, 2007). To shed light on superoxide production during OHT, we performed *in situ* detection of the oxidation product of dihydroethidium, and investigated the levels and activity of NOX-2 by qPCR, Western blotting, and immunohistochemistry.

**Figure 10:**
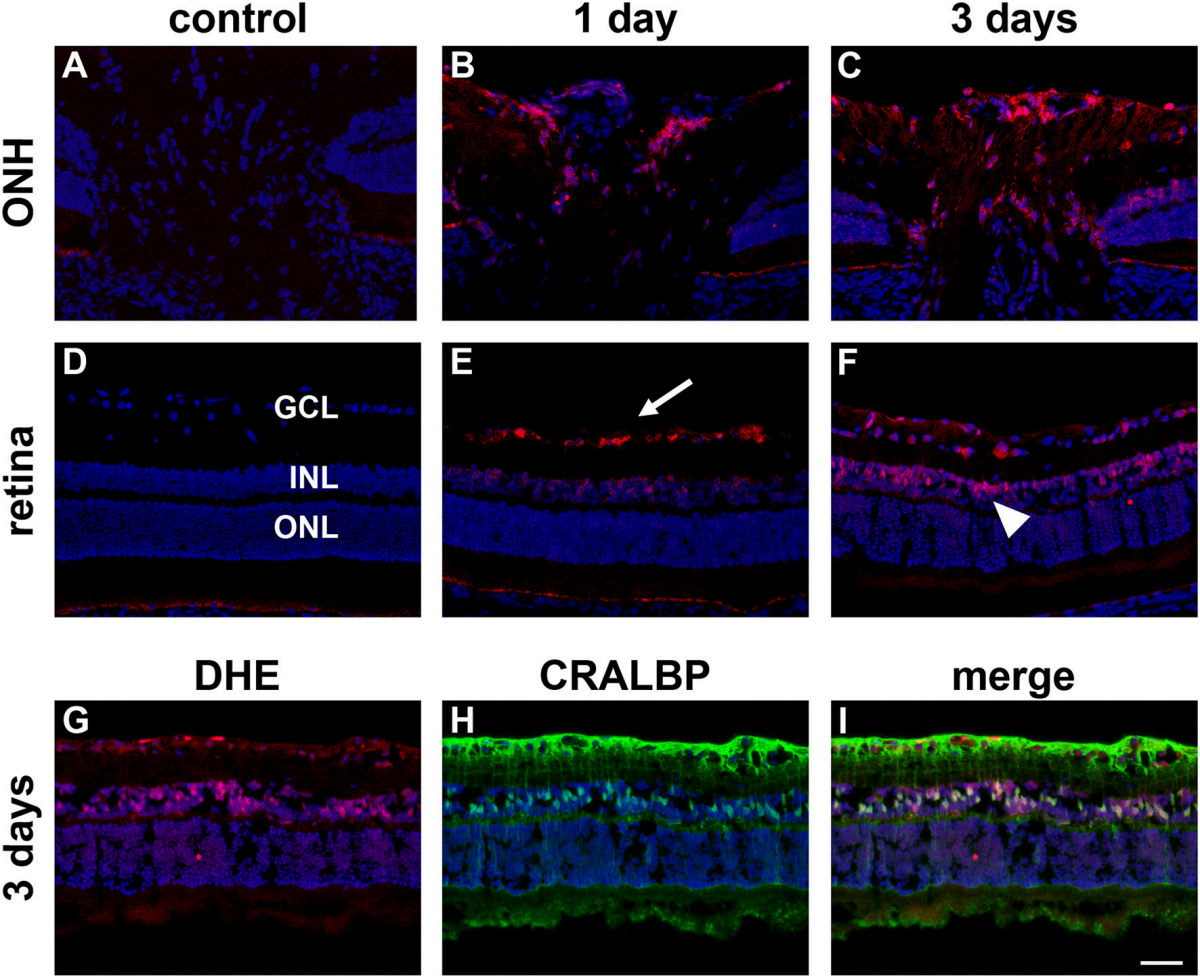
Increased superoxide production in the retina and prelaminar optic nerve head (ONH) after induction of ocular hypertension (OHT). **(A–F)** Representative images of sections of the prelaminar ONH and retina incubated with dihydroethidium (DHE, red) from a control eye, and from eyes subjected to ocular hypertension for 1 and 3 days. Sections are counterstained with the nuclear dye DAPI. Negligible fluorescence is evident in sections from the control. In contrast, after 1 day of OHT, cells residing within the retinal ganglion cell layer (GCL, arrow), some cells within the inner nuclear layer (INL), and glial cells at the ONH display DHE fluorescence. After 3d of OHT, DHE staining is more prominent, notably by cells in the INL (arrowhead) and at the ONH. ONL, outer nuclear layer. **(G–I)** Double labeling immunofluorescence of DHE (red) with the Müller cell marker CRALBP (green) at 3 days after induction of OHT. Scale bar: 50 μm.

In control eyes, superoxide formation, as determined by oxidation of dihydroethidium, was barely detectable (Figures 10A,D). After 1 d of OHT, RGCs throughout the retina and glial cells at the prelaminar ONH were prominently stained, whilst some Müller cells and axonal bundles in the central nerve fiber layer displayed fainter staining (Figures 10B,E). After 3 d of OHT, a similar pattern of staining was observed, although by this later time point both Müller cell and axonal staining were stronger (Figures 10C,F). Confirmation that superoxide staining within the retinal inner nuclear layer principally reflected Müller cells was achieved by double labeling with CRALBP (Figures 10G–I). All four animals at each time point displayed analogous patterns of fluorescence. The protocol used for determination of superoxide detection entailed intravitreal injection of dihydroethidium. As such, penetration of the compound to the optic nerve was likely highly inadequate and the laminar region of the ONH stained only faintly. For this reason, and also owing to the low “*n*” numbers, quantification was not carried out.

**Figure 11:**
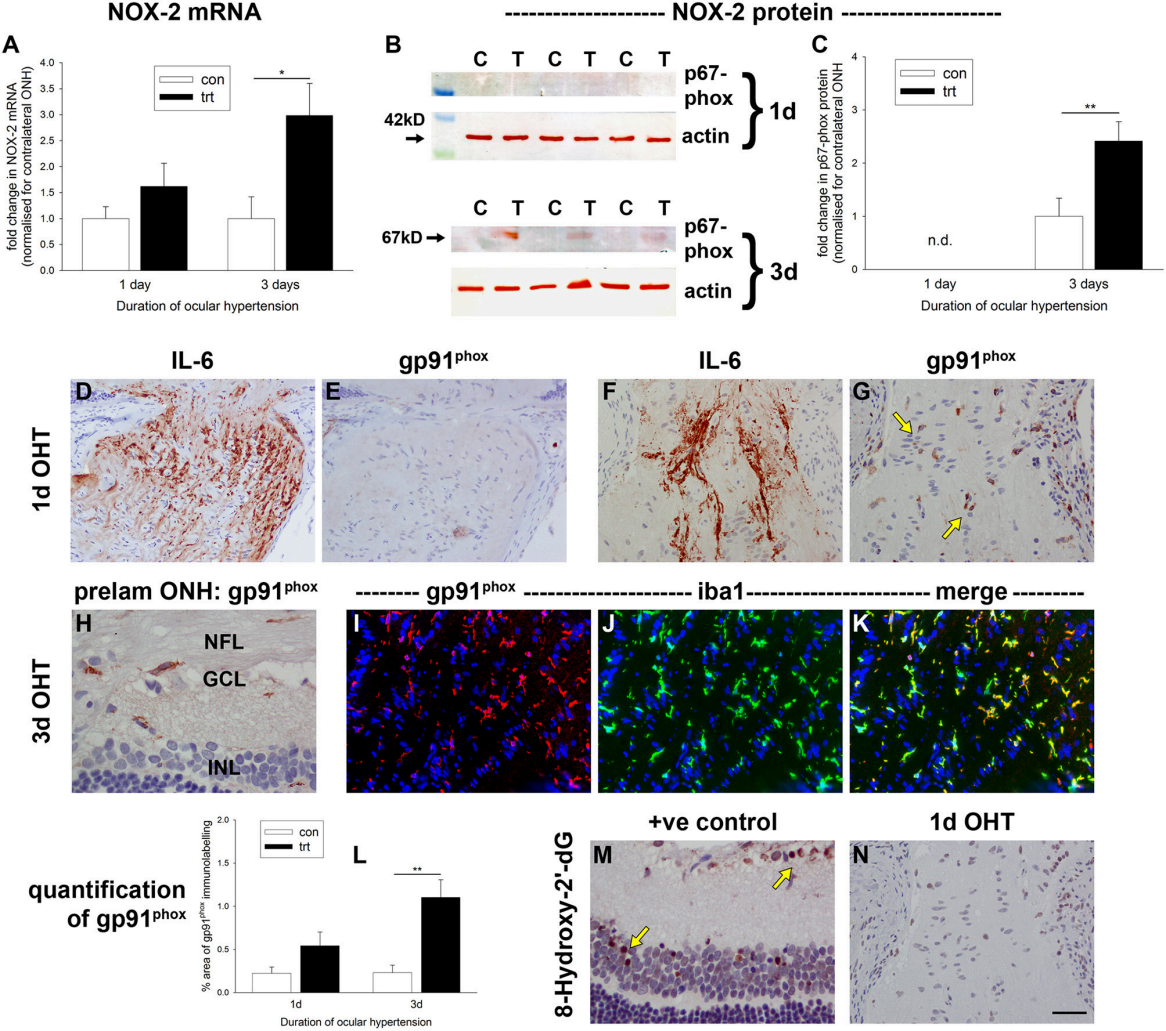
Evaluation of NADPH oxidase-2 (NOX-2) and 8-hydroxy-2′-deoxyguanosine in the optic nerve head (ONH) after induction of ocular hypertension (OHT). **(A)** Quantification of NOX-2 mRNA in ONH extracts, as measured by qPCR. Data are normalized for rhodopsin and expressed relative to controls. Values represent mean ± SEM, where *n* = 12–14. ^*^P < 0.05, by Student's unpaired *t-*test. **(B,C)** Expression of the regulatory subunit for NOX-2, p67^phox^, in optic nerve head (ONH) extracts at 1 and 3 d after induction of ocular hypertension, as determined by Western immunoblotting. Representative immunoblots from three animals at each time point are shown (C, control ONH; T, treated ONH). Single bands of the expected molecular weight are apparent. β-actin immunoblots for the same samples are also shown, as gel-loading controls. Values (represented as mean ± SEM, where *n* = 12–14) are normalized for actin and expressed relative to controls. ^**^P < 0.01, by Student's unpaired *t-*test. **(D–K)** Representative images of gp91^phox^ (the catalytic subunit of NOX-2) immunolabeling in tissue sections from control and OHT eyes. **(D–G)** After 24 h of OHT, gp91^phox^-positive cells (**G**, arrows) are evident in ONHs from some OHT rats, but not in others **(F)**. The presence of gp91^phox^ expression appears not to be related to the exent of axonal transport disruption, shown alongside for comparative purposes **(D,F)**. **(H–K)** After 3 d of OHT, all OHT eyes feature gp91^phox^-positive cells, throughout the prelaminar **(H)** and laminar ONH **(I–K)**. Double labeling immunofluorescence of gp91^phox^ (red) with iba1 (green) reveals an almost complete co-localization in microglia **(I–K)**. **(L)** Quantification of gp91^phox^ immunolabeling in the ONH. Values represent mean ± SEM, where *n* = 12–16. ^**^P < 0.01, by Student's unpaired *t-*test. **(M,N)** Representative images of 8-hydroxy-2′-deoxyguanosine (8-hydroxy-2′-dG) immunolabeling in the retina following injection of the excitotoxic glutamatergic agonist, N-methyl-D-aspartate, and in the ONH (also shown in **G**) after 24 h of OHT. 8-Hydroxy-2′-dG-positive cells are numerous in the positive control specimen, but not within the ONH of OHT rats. Scale bar: **(D–G,N)** = 50 μm; **(H–K,M)** = 25 μm.

qPCR data of dissected ONH samples revealed increases in NOX-2 mRNA in the treated vs. the untreated, contralateral eye of ~1.5-fold at 1 d, and three-fold at 3 d, after induction of OHT (Figure 11A). This difference only reached statistical significance (*P* = 0.13 at 1 d; *P* < 0.05 at 3 d) at the latter time point. Western blotting analysis of the regulatory subunit of NOX-2, p67^phox^, revealed a similar trend: the protein was barely detectable in control ONH extracts, was unchanged at 1 d after induction of OHT, but was significantly elevated after 3 d of OHT (*P* < 0.01; Figures 11B,C). To provide a more informative spatial perspective, as well as insight into activity, we immunolabeled tissue sections with an antibody directed against the catalytic subunit of NOX-2, gp91^phox^. In control eyes, occasional gp91^phox^-positive cells were detected (data not shown). After 1 d of OHT, some ONH sections displayed numerous gp91^phox^-positive cells, others featured occasional faintly-labeled cells, while other ONHs had no discernible gp91^phox^ immunolabeling (Figures 11E,G). Surprisingly, the expression of gp91^phox^ did not reflect the abundance of axonal transport disruption (Figures 11D,F). Double labeling with the macrophage/microglial-specific marker iba1 indicated that expression of gp91^phox^ was restricted to this cell type (data not shown). By 3 d of OHT, gp91^phox^-positive microglia were observed in all ONH sections and also within the inner retina (Figures 11H–K, Supplementary Figure 4), whilst gp91^phox^-positive macrophages were prominent at the site of laser trabeculoplasty, signifying inflammation at this location (Supplementary Figure 4). Quantification of the area of gp91^phox^ immunolabeling in images taken from the ONH (Figure 11L) revealed a significant increase within this region at 3 d (*P* < 0.01), but not 1 d (*P* = 0.08).

### Ocular hypertension does not appear to be associated with oxidative DNA damage in the optic nerve head

Oxidative stress leads to DNA, protein and lipid damage. The guanine base in DNA or RNA is oxidized by ROS and changes to 8-hydroxy-2′-deoxyguanosine, which can be monitored immunohistochemically. 8-Hydroxy-2′-deoxyguanosine, thus, serves as a useful measure of oxidative stress. We analyzed ONH tissue sections from 1 and 3 d OHT eyes, but at neither of the time points did we detect any positive labeling for -hydroxy-2′-deoxyguanosine, even in ONH sections with numerous gp91^phox^-positive cells and axonal injury (Figure 11N). The validity of the assay was proven by positive staining in eyes injected with the excitotoxic glutamatergic agonist, N-methyl-D-aspartate (Figure 11M).

## Discussion

Failure of orthograde axonal transport at the ONH has been hitherto identified as the earliest pathological event following chronic elevation of IOP. Analogous results have been found in primates (Anderson and Hendrickson, 1974; Quigley and Anderson, 1976, 1977; Minckler et al., 1977) and pigs (Balaratnasingam et al., 2007), which possess a collagenous lamina cribrosa, as well as rodents (Howell et al., 2007; Salinas-Navarro et al., 2010; Chidlow et al., 2011b), which feature an astrocyte-rich glial lamina (Sun et al., 2009). The underlying cause of axonal injury at the ONH in response to OHT is unknown, but conceivably involves bioenergetic deficiency resulting from a decreased oxygen/nutrient supply (Inman and Harun-Or-Rashid, 2017). In the current study, we have employed a rat model of OHT that features reproducible and widespread axonal transport disruption at the ONH by 24 h after chronic elevation of IOP (Salinas-Navarro et al., 2010; Chidlow et al., 2011b). Using a range of immunohistochemical and molecular tools, we looked for cellular events that may be indicative of vascular insufficiency.

Initially, we examined whether OHT-induced axonal transport disruption is spatially and temporally associated with oxygen deprivation. The hypoxia marker pimonidazole binds cells with an oxygen tension of <10 mmHg (Arteel et al., 1995), and has been successfully employed to reveal hypoxia within the optic nerve in a rat model of anterior ischemic optic neuropathy (Danylkova et al., 2006), and, within the retina in a murine model of oxygen-induced retinopathy (Gardiner et al., 2005; Mowat et al., 2010) and in rats subjected to acute elevation of IOP (Holcombe et al., 2008). Our results revealed some hypoxic staining within the laminar ONH in 11/13 eyes graded as either medium or high for axonal transport disruption. Pimonidazole always labeled in areas of the ONH featuring injured axons, and, the greater the abundance of axonal transport disruption, the greater the likelihood of a larger hypoxic region. Nevertheless, hypoxic regions were typically focal and were not necessarily evident in sections taken deeper within the same ONH, while disrupted axonal transport was frequently encountered without any pimonidazole labeling, and, hypoxia was not evident in animals graded as low for axonal transport disruption. Overall, it can be concluded that there is a negative effect of moderate, chronic OHT upon ONH tissue oxygenation, coinciding with axonal transport failure, but that the deficit in oxygen is not uniform throughout the ONH. It is reasonable to postulate the existence of areas of non-, or low, perfusion in watershed zones in the ONH. In future, it may be worth analyzing serial sections through the entire ONH for hypoxia and constructing 3-dimensional models. It is a weakness of the current study that only three levels of each ONH were analyzed for the presence of hypoxia.

In the cat, oxygen tension within the prelaminar ONH has been reported to exist within the range of 10–32 mm Hg (Shonat et al., 1992; Ahmed et al., 1994). Acute elevation of IOP to ~40 mm Hg—a level very similar to that measured in our “high injury” cohort—yielded negligible impact upon oxygen tension within the prelaminar ONH or retina (Shonat et al., 1992; Ahmed et al., 1994), indicating a system with effective autoregulation. As such, we might not have expected to detect hypoxia at the ONH in the current study. A number of factors might account for the apparent dissimilarity: firstly, we only observed pimonidazole staining within the laminar, not the prelaminar, ONH. To knowledge, oxygen tension within the laminar ONH in response to elevation of IOP has not been reported and may differ from the prelaminar region; secondly, autoregulation may not function as efficiently during prolonged OHT as compared to acute OHT; thirdly, the ONH vasculature of the cat differs considerably from that of the rat. Despite the low oxygen tension present in the inner retina of the healthy rat eye (Yu and Cringle, 2001), chronic OHT did not give rise to noteworthy hypoxia in the retina, with staining limited to occasional pimonidazole-positive cells in the ganglion cell layer. These results essentially reflect those previously reported following acute elevation of IOP, where RGC hypoxia was not observed below an IOP of 70 mm Hg (Holcombe et al., 2008). Our results show that autoregulation maintains perfusion to the rat retina reasonably effectively even during OHT.

We did not assess vascular damage in the current study, as we considered it unlikely as such early time points prior to axon loss. In humans and monkeys with glaucoma, capillary drop out within the optic disc has been shown to occur in proportion to axon loss: as fibers atrophy, so do capillaries. Thus, capillaries are not disproportionately lost early in glaucoma (Quigley et al., 1984). Such results match those of Valiente-Soriano et al. (2015), who found no abnormalities in the inner retinal vasculature after 2 weeks of raised IOP that could account for the sectorial loss of RGCs. As regards microvascular function, it has been argued that raised IOP compresses capillaries, reducing blood supply to axons, without causing actual capillary loss. There is no strong evidence from fluorescein angiography that defects at the ONH substantially precede neuron loss, but the great difficulty, as noted by Quigley et al. (1984), is that the capillaries of greatest interest in glaucoma pathogenesis are hidden from clinical and angiographic view. In our study, hypoxia was not detected at the superficial ONH, only within the laminar region. As such, localized sub-optimal microvascular perfusion remains a likely explanation for the observed hypoxia. As a final point, rats in our study with a lower magnitude pressure rise tended not to have detectable ONH hypoxia; thus, it might be predicted that elevation of IOP for long periods of time may likewise not produce hypoxia; however, longer term moderate ocular hypertension may slowly damage ONH capillaries, eventually resulting in hypoxia. Moreover, rats in this study were young and ostensibly healthy; elevation of IOP in older humans may conceivably elicit hypoxia at lower pressures owing to a diminished abililty of capillaries to withstand any mechanical stress.

We next investigated expression of HO-1, the inducible, rate-limiting enzyme in heme catabolism. HO-1, a gene target of both HIF-1 and Nrf2, is one of the most studied genes in conditions of hypoxia and oxidative stress and is believed to play an important role in the endogenous response of tissues to oxidative injuries (Ryter et al., 2006; Jazwa and Cuadrado, 2010). Our results showed a robust and consistent induction of HO-1 by astrocytes within the ONH after 1 d of OHT. The distribution and abundance of HO-1 closely matched that of axonal transport disruption. HO-1 expression encompassed hypoxic regions and their immediate penumbra, but was also observed in ONH sections of pimonidazole rats that did not feature an overt hypoxic region, indicating that HO-1 is a sensitive and early glial-expressed marker of axonal injury during OHT. At present, it is unclear whether HO-1 within the ONH is upregulated in response to lower-than-normal oxygen tension or a higher-than-normal concentration of ROS. In cultured brain astrocytes, both hypoxia (Kuwabara et al., 1996; Imuta et al., 2007) and exogenously-applied oxidative stress (Lee et al., 2003) have been shown to induce HO-1 expression. Of particular relevance to glaucoma, oxidative stress also upregulates HO-1 expression in cultured ONH astrocytes (Yu et al., 2009; Noh et al., 2013) and cultured retinal astrocytes (Nahirnyj et al., 2013). Contrary to the unambiguous results of this study, HO-1 mRNA was not found to be upregulated in ONH extracts taken from rats with early or advanced optic nerve injury profiles in the hypertonic saline model of OHT (Johnson et al., 2011). An explanation for the differing results likely relates to the chronology of IOP elevation, axonal injury and HO-1 mRNA induction in the OHT model used. While neuronal injury can lead to a prolonged upregulation in HO-1 protein, the mRNA itself is typically upregulated for only a short time window.

In contrast to the ONH, HO-1 was not widely expressed by retinal glial cells until 3 d after induction of OHT, a time point coincident with a demonstrably increased signal for the superoxide-sensitive marker DHE. Interestingly, RGCs displayed strong labeling for DHE after just 1 d of OHT, but no HO-1 immunolabeling was evident in the GCL until 3 d after induction of OHT, and even then only in occasional cells. The results are consistent with those of brain studies, for example Dwyer et al. (1995), who compared HO-1 induction in cultured cortical neurons and forebrain astrocytes following an oxidative stress challenge and showed that in spite of increased generation of free radicals in neurons, the HO-1 protein level was relatively unchanged, whilst it was upregulated seven-fold within a few hours in the astrocyte cultures. The efficacy of astrocytes may be a factor in their own survival, and that of neighboring neurons, in the face of oxidative challenge. Indeed, the consensus reached from a wealth of studies is that acute induction of HO-1 by oxidative stress is an adaptive mechanism that controls the severity of neuronal damage. Over-expression or pharmacological induction of HO-1 is neuroprotective in numerous models of CNS injury (Schipper et al., 2009; Jazwa and Cuadrado, 2010), including retinal ischemia-reperfusion (Peng et al., 2008; Sun et al., 2010), optic nerve crush (Himori et al., 2014), and diabetic retinopathy (Fan et al., 2012). The products of HO-1 activity have potent antioxidant and anti-inflammatory properties that are thought to mediate the observed neuroprotection. Future studies should investigate whether overexpression of HO-1 is neuroprotective in experimental glaucoma.

HO-1 is one of many oxygen-regulated genes involved in the adaptative response of cells to hypoxia. Other iron metabolism genes, such as ceruloplasmin, transferrin receptor and transferrin, have also been implicated (Chepelev and Willmore, 2011), alongside genes encoding glucose transport and glycolytic enzymes (Wenger, 2002). We found increased levels of LDH-A and ceruloplasmin in the hypertensive ONH concurrent with axonal transport disruption, HO-1 expression and pimonidazole staining. The results are in agreement with previous work showing that brain astrocytes upregulate glycolytic genes, including LDH-A, in response to hypoxia (Marrif and Juurlink, 1999; Mense et al., 2006). As a note of caution, it should be acknowledged that analyzing LDH-A expression is less informative than studying enzyme activity or lactate levels in terms of demonstrating anaerobic glycolysis. Our finding of upregulated ceruloplasmin likewise corresponds with previous reports demonstrating increased expression of ceruloplasmin in murine, primate and human glaucomatous retinas (Farkas et al., 2004; Stasi et al., 2007).

Hypoxia-induced modification of gene expression is not solely mediated via HIF-1. Other transcription factors, notably AP-1—a dimer composed of JUN/FOS or JUN/JUN subunits—can also be activated by hypoxia (Cummins and Taylor, 2005). Dimerization between JUN/FOS subunits as well as phosphorylation of either JUN or FOS is required for DNA-binding and transcriptional activity of AP-1. In the current study, we observed a striking induction of both cFos and the phosphorylated form of cJun by astrocytes within the ONH after 1 d of OHT. An identical response was previously reported in monkeys with longer-term experimental glaucoma (Hashimoto et al., 2005). AP-1 is a redox-sensitive transcription factor, thus, increased ROS rather than hypoxia may be the stimulus for its activation; nevertheless, this finding underscores the rapid response of ONH astrocytes to perturbations in homeostasis.

Any hypoxia or nutrient deficiency in the ONH (resulting from vascular insufficiency) during OHT will likely result in impaired mitochondrial functioning, an increased production of ROS, and eventually, in oxidative stress-induced cell damage (Chrysostomou et al., 2013). In an attempt to safeguard homeostasis from oxidative stress, cells have evolved the means to upregulate antioxidant genes under the control of the redox-sensitive transcription factor Nrf2 (Tebay et al., 2015). Astrocytes in the ONH occupy up to one half of the tissue volume—a much greater percentage than in the myelinated optic nerve (Skoff et al., 1986)—and they likely have a significant influence upon the progression of glaucoma, whether supporting axonal survival or contributing to neuroinflammation (Soto and Howell, 2014; Williams et al., 2017). Recent studies using cultured ONH astrocytes have demonstrated that exogenously-administered oxidative stress fortifies their antioxidant defenses, resulting in elevated levels of Nrf2, SOD-2, HO-1, and Hsp27 (Malone and Hernandez, 2007; Yu et al., 2008, 2009; Noh et al., 2013). In the current study, we undertook preliminary analysis of ROS formation and antioxidant defenses at 1 d after induction of OHT when axonal transport disruption at the ONH is maximal, and, following 3 d of OHT when axonal degeneration has commenced. We found a significant upregulation of Nrf2—the master regulator of antioxidant defenses (Tebay et al., 2015)—in ONH extracts, but not of other well-described antioxidant enzymes, such as NQO1, GCLM, Prdx6, or SOD-1. Prdx6, which is expressed exclusively by astrocytes in the ONH (Chidlow et al., 2016), was of particular interest given the finding of increased expression in reactive astrocytes following ischemia-reperfusion in the hippocampus (Zhang et al., 2013). Moreover, we failed to detect any oxidative DNA damage to astrocytes at either time point. While these results are suggestive of a lack of detrimental oxidative stress within ONH astrocytes concurrent with axonal transport injury, it should be noted that only modest inductions of antioxidant genes were reported in primary cortical astrocyte cultures subjected to oxidative stress (Lee et al., 2003). Moreover, we did observe an increased level of superoxide in ONH astrocytes, as well as a consistent elevation of the mitochondrial isoform of superoxide dismutase (SOD-2) in ONH extracts. Since SOD-2 is present in all cells with mitochondria, and is abundant in non-myelinated axons in the ONH, it was not feasible to delineate whether the increased signal reflected any upregulation by astrocytes. Interestingly, RGC somas also displayed increased superoxide after 1 d of OHT. This finding matches the earlier work of Kanamori et al. (2010), who identified superoxide labeling of RGC somas at 1 d after optic nerve transection, and suggested that this represented an upstream signal for RGC apoptosis following axonal injury.

While the mitochondrial respiratory chain is the major contributor to excessive ROS, the dedicated superoxide-generating enzyme NADPH oxidase (NOX), in particular the phagocytic NOX-2 isoform, is increasingly being implicated as a source of detrimental ROS in acute and chronic neurodegenerative conditions (Ma et al., 2017). We postulated that ONH glia, chiefly microglia, triggered by elevation of IOP, upregulate NOX-2, and release a burst of superoxide, which contributes to axonal transport failure. In fact, our data show that there was no consistent pattern of NOX-2 expression after 1 d of OHT, concomitant with axonal transport disruption. It must be concluded that NOX-2 does not contribute significantly to initial axonal injury. Expression of NOX-2 by ONH microglia was, however, widespread after 3 d of OHT. Presumably, this response contributes to phagocytosis of injured axons. Future work should determine whether inhibition of NOX-2 augments axonal survival in glaucoma, as has been shown to be the case in numerous other neurodegenerative conditions.

The overall results of this study provide tentative support for the hypothesis that reduced blood flow to the ONH contributes to RGC axonal injury; however, the data cannot be viewed as providing direct evidence for a causative relationship between hypoxia—or cellular events downstream of hypoxia—and axonal transport failure. Future work needs to identify if reducing the existence, or even the extent, of hypoxia, or if augmenting energy availability and/or antioxidant defenses prevents the axonal transport failure that occurs during OHT.

## Author contributions

All authors had full access to all the data in the study and take responsibility for the integrity of the data and the accuracy of the data analysis. Study concept and design: GC, JW, and RC. Acquisition of data: GC and JW. Analysis and interpretation of data: GC and JW. Drafting of the manuscript: GC. Critical revision of the manuscript for important intellectual content: JW and RC. Statistical analysis: GC and RC. Obtained funding: GC. Administrative, technical, and material support: RC.

### Conflict of interest statement

The authors declare that the research was conducted in the absence of any commercial or financial relationships that could be construed as a potential conflict of interest. The reviewer DI and handling Editor declared their shared affiliation.
