# Supplementary material for: Investigations into Hypoxia and Oxidative Stress at the Optic Nerve Head in a Rat Model of Glaucoma
Source: Front Neurosci. 2017 Aug 24;11:478. doi: 10.3389/fnins.2017.00478 (PMC5573812; doi:10.3389/fnins.2017.00478)

**Supplementary Figure 1**. (**A**-**C**) Double labeling of heme oxygenase-1 (HO-1) with vimentin within the optic nerve head (ONH) at 1d following induction of ocular hypertension. Co-localization of HO-1 is observed in vimentin-positive astrocytes. (**D**-**K**) Representative images of HO-1 immunolabeling in ONH and retinal tissue sections at 3d after induction of OHT. By 3d, HO-1 is upregulated by ONH glial cells with the morphological appearance of microglia, in addition to astrocytes (**D**, **E**). In the retina, HO-1 immunolabelling is evident in microglia cells in the nerve fiber layer (arrows), GCL and inner plexiform layer (**F**, **G**). Occasional RGCs (arrowheads) express HO-1 (**H, I**), while Müller cell process are also frequently HO-1-positive (**J**, **K**). GCL, ganglion cell layer; INL, inner nuclear layer. Scale bars: A-C = 25μm; D-K = 25μm.


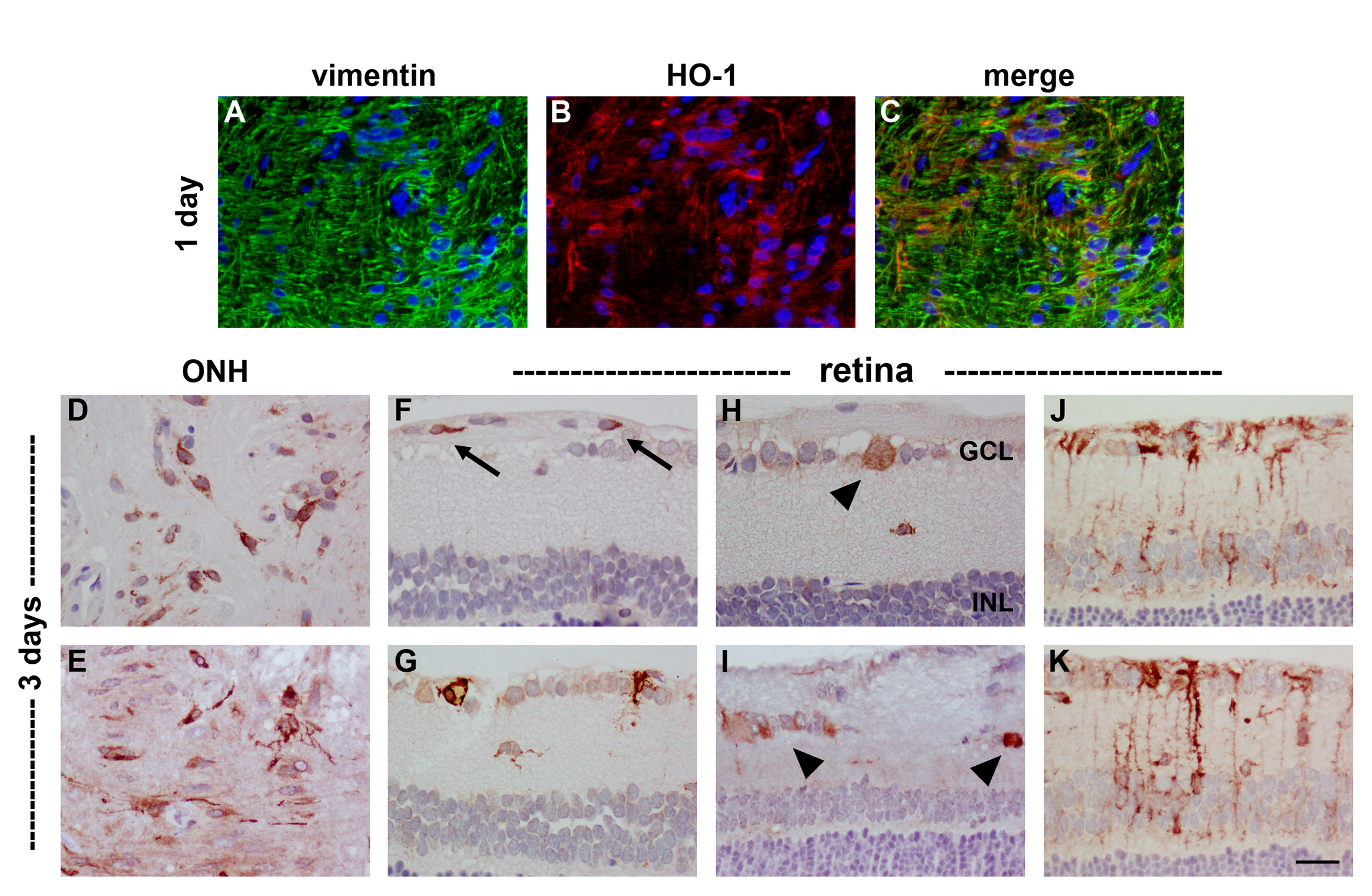

Supplement: Supplementary file 1 [file DataSheet1.doc]
