# Supplementary material for: Investigations into Hypoxia and Oxidative Stress at the Optic Nerve Head in a Rat Model of Glaucoma
Source: Front Neurosci. 2017 Aug 24;11:478. doi: 10.3389/fnins.2017.00478 (PMC5573812; doi:10.3389/fnins.2017.00478)

**Supplementary Figure 2**. (**A**-**C**) Localization of transferrin receptor (TR) within the control optic nerve. TR immunolabeling is associated with glial cells with the morphology of oligodendrocytes and with capillary endothelial cells (arrows). (**D**-**F**) Localization of ceruloplasmin (cerulo) within the control optic nerve head (ONH) and optic nerve (ON). Ceruloplasmin immunolabelling is distributed in a pattern characteristic of astrocytes. Scale bars: A-C = 25μm; D-F = 50μm.


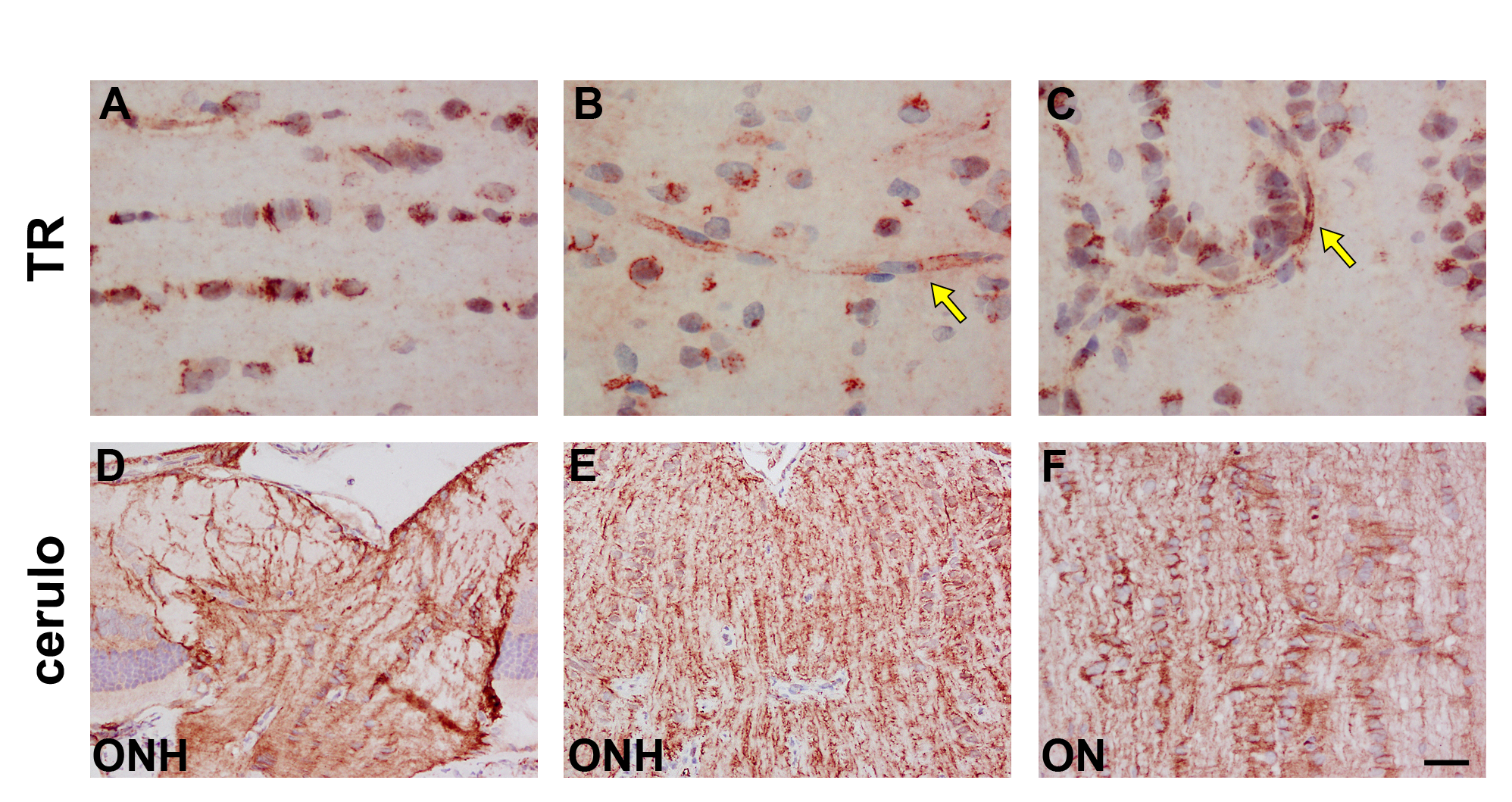

Supplement: Supplementary file 2 [file DataSheet2.DOC]
