# Supplementary material for: Investigations into Hypoxia and Oxidative Stress at the Optic Nerve Head in a Rat Model of Glaucoma
Source: Front Neurosci. 2017 Aug 24;11:478. doi: 10.3389/fnins.2017.00478 (PMC5573812; doi:10.3389/fnins.2017.00478)

**Supplementary Figure 3**. Double labeling of p-cJun and cFos with the astrocytic marker S100 in the ONH at 1d following induction of ocular hypertension. (**A**-**C**) Double labeling of p-cJun (red) with S100 (green). (**D**-**F**) Double labeling of cFos (red) with S100 (green). Both p-cJun and cFos colocalise with S100 (arrows). Scale bar: 50μm.


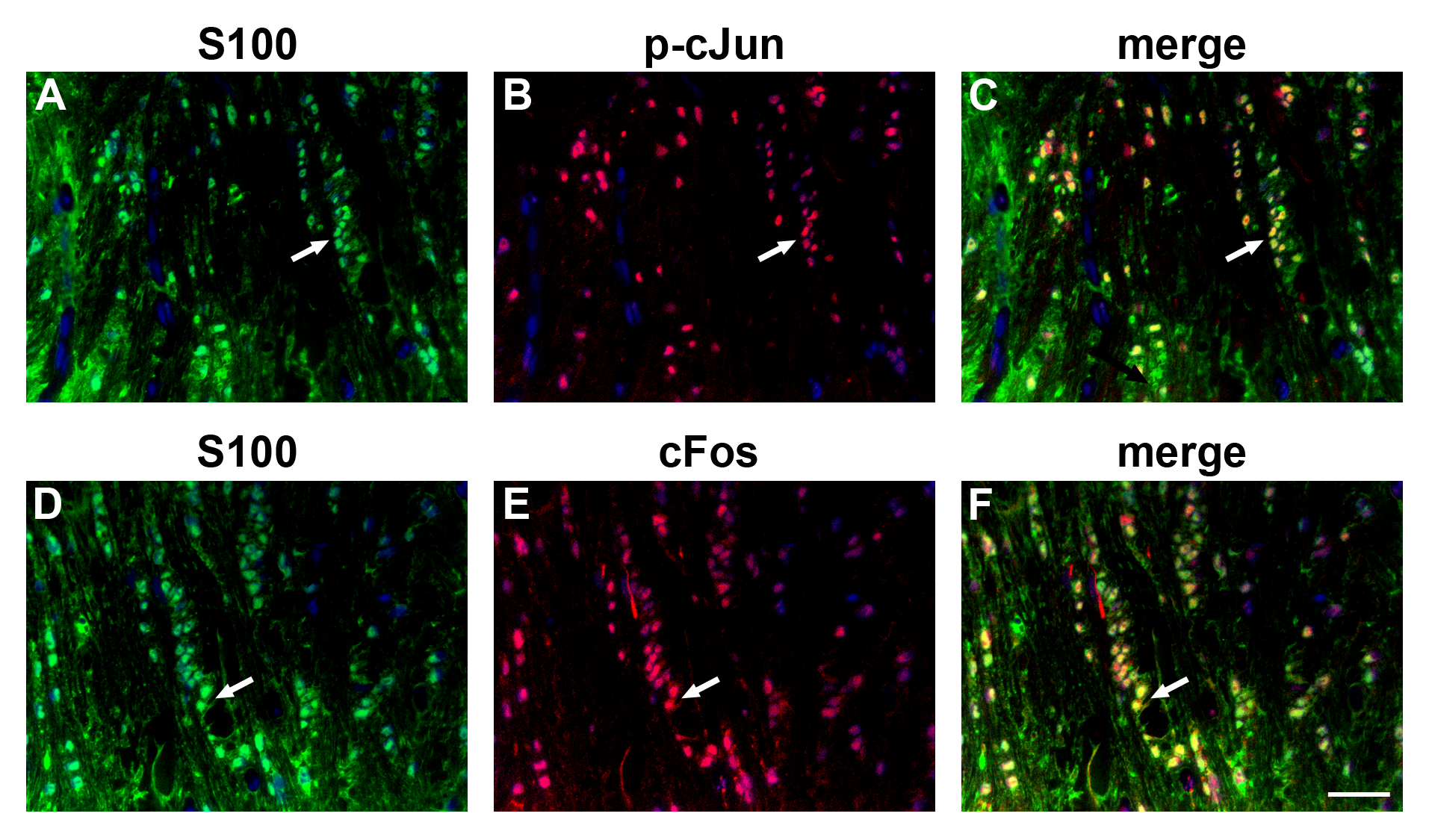

Supplement: Supplementary file 3 [file DataSheet3.DOC]
