# Supplementary material for: Investigations into Hypoxia and Oxidative Stress at the Optic Nerve Head in a Rat Model of Glaucoma
Source: Front Neurosci. 2017 Aug 24;11:478. doi: 10.3389/fnins.2017.00478 (PMC5573812; doi:10.3389/fnins.2017.00478)

**Supplementary Figure 4**. Double labeling of gp91^phox^ (red) with iba1 (green) at 3d following induction of ocular hypertension. (**A**-**C**) Localization of gp91^phox^-positive macrophages at the site of laser trabeculoplasty (anterior chamber angle). (**D**-**F**) C-localization of gp91^phox^-positive cells with iba1-positive microglia within the prelaminar and laminar optic nerve head (ONH). Scale bar: 50μm.


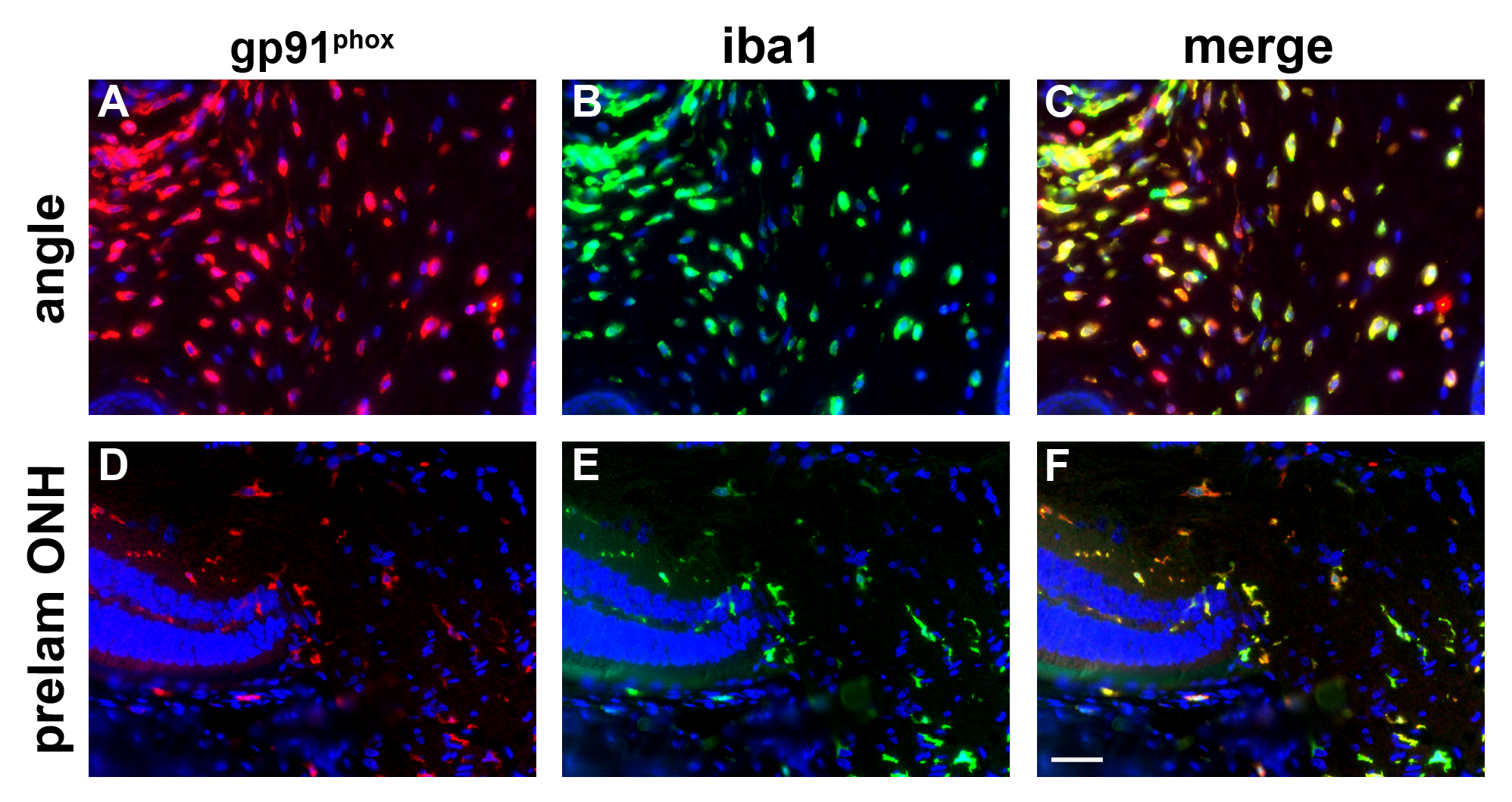

Supplement: Supplementary file 4 [file DataSheet4.DOC]
